# Gut Commensal Antibiotic-Resistant *Parabacteroides goldsteinii* Ameliorates Mouse Colitis through Valine–Isobutyrate Metabolism

**DOI:** 10.34133/research.0867

**Published:** 2025-09-11

**Authors:** Ningning He, Mengjie Mu, Xiaofang Li, Qingyuan Hao, Kaiwei Chen, Xinnan Zhao, Yang Sun, Haoyu Wang, Zhinan Wu, Hewei Liang, Mengmeng Wang, Liang Xiao, Tao Yu, Zhi-Peng Wang, Jixing Peng, Yuanqiang Zou, Shangyong Li

**Affiliations:** ^1^ School of Basic Medicine, Qingdao Medical College, Qingdao University, Qingdao 266071, China.; ^2^ BGI Research, Shenzhen 518083 China.; ^3^ State Key Laboratory of Genome and Multi-omics Technologies, BGI Research, Shenzhen 518083, China.; ^4^ Yellow Sea Fisheries Research Institute, Chinese Academy of Fishery Sciences, Qingdao 266071, China.; ^5^ Gastroenterology, The First Affiliated Hospital of Kunming Medical University, Kunming 650032, China.; ^6^ College of Life Sciences, University of Chinese Academy of Sciences, Beijing 100049, China.; ^7^ Shenzhen Key Laboratory of Bioenergy, BGI Research, Shenzhen 518083, China.; ^8^ BGI Precision Nutrition (Shenzhen) Technology Co., Ltd, Shenzhen 518083, China.; ^9^ School of Marine Science and Engineering, Qingdao Agricultural University, Qingdao 266109, China.

## Abstract

Antibiotic cocktails (ABX) serve as potent therapeutic interventions for refractory ulcerative colitis (UC), yet invariably induce gut dysbiosis. This study demonstrates that pectin oligosaccharides synergistically enhance ABX efficacy by restoring gut microbiota balance and selectively enriched antibiotic-resistant *Parabacteroides goldsteinii* in a colitis mouse model. Our results further indicate that the gavage administration of *P. goldsteinii* AM58-2XD markedly alleviated colitis via enhancing the branched-chain amino acid metabolic pathway, particularly by facilitating valine metabolism. Notably, these anticolitis effects were partially attenuated in *P. goldsteinii*^ΔilvE^ mutants, which are defective in valine-derived isobutyrate (IBN) biosynthesis. We further demonstrated that exogenous IBN supplementation effectively alleviated colitis symptoms in mice and enhanced gut barrier function via activation of the peroxisome proliferator-activated receptor γ (PPARγ) pathway. Conditional knockout of PPARγ in Caco-2 intestinal epithelial cells markedly abrogated the IBN-induced enhancement of tight junctions, thereby substantiating the critical role of the IBN-PPARγ pathway in metabolite-mediated mucosal repair. Collectively, we delineate a prebiotic/probiotic–metabolite axis wherein *P. goldsteinii* facilitates mucosal repair via IBN/PPARγ-dependent epithelial metabolic reprogramming. This insight redefines antibiotic-resistant commensals as precise biotherapeutics for microbiota restoration in refractory UC management.

## Introduction

Ulcerative colitis (UC) is a chronic inflammatory bowel disease of the colon’s mucosa, characterized by clinical manifestations including bloody and mucoid diarrhea, abdominal pain, and rectal bleeding [1,2]. The etiology of UC is intricate, including genetics, environmental factors, and immune system disorders [3–5].While broad-spectrum oral antibiotics (ABX) show promise in treating acute severe and chronic refractory UC, their clinical application remains limited primarily due to their inevitable induction of gut microbiota dysbiosis, unknown long-term sequela, and compromise of intestinal barrier integrity [6,7]. The reconstitution process of post-antibiotic microbiome is frequently slow and variable [8]. Moreover, the emergence of antibiotic-resistant bacterial strains, both commensal and pathogenic, poses a significant clinical threat, especially in patients with recurrent UC flares requiring repeated antibiotic exposure [9]. A recent study has highlighted that the gut microbiome of UC patients tends to harbor increased antibiotic resistance gene signatures compared to healthy individuals [10], suggesting that indiscriminate ABX use may further exacerbate microbial dysbiosis and therapeutic inefficacy.

Accumulating evidences have demonstrated that various prebiotics and probiotics exhibit preventive and ameliorative effects on UC via modulating gut microbiota dysbiosis and protecting intestinal barrier integrity [11–14]. Meanwhile, another important usage of prebiotics and probiotics is the occurrence of diarrhea associated with UC or antibiotic administration [15]. They serve as viable preventive measures against gut dysbiosis and associated complications induced by antibiotic therapy in murine models and human studies [16–18]. However, there is limited research on the relationship between prebiotic intake and antibiotics, as well as how probiotics affect the intestinal mucosal barrier through metabolic pathways.

*Parabacteroides goldsteinii*, a gut commensal bacterium, has been identified as a promising probiotic candidate owing to its multifaceted role in ameliorating metabolic disorders and inflammatory conditions [19,20]. Recent research have indicated that *Parabacteroides* spp., including *P. goldsteinii*, are increasingly exhibiting resistance to certain antibiotics, likely due to the enrichment of virulence-associated genes linked to antimicrobial resistance within this genus [21]. Mice were treated with multiple antibiotics, and the relative abundance of *P. goldsteinii* varied among different antibiotic-treated groups [22]. This may imply that *P. goldsteinii* has a certain degree of tolerance to these antibiotics, or its ecological adaptability under the action of antibiotics leads to changes in its abundance. However, the direct causal relationship between *P. goldsteinii* and UC pathogenesis, as well as its mechanistic interplay with antibiotics and dietary factors, remains unresolved. Our previous study in mice with dextran sulfate sodium (DSS)-induced colitis revealed the preventive and prebiotic effects of pectin oligosaccharides (POS) [23]. However, it remains unclear whether POS exhibits a synergistic therapeutic effect when combined with antibiotics, and which specific species are enriched and play key contributors, as well as the underlying mechanism between host and gut microbiota.

In this study, we investigated the synergistic therapeutic efficacy of POS in combination with ABX (POS+ABX) therapy in mice with DSS-induced colitis. Meanwhile, we found that *P. goldsteinii* is the key contributor of POS+ABX therapy in colitis mice partially through valine–isobutyrate (IBN) metabolism. Our findings further highlight the therapeutic potential of *P. goldsteinii*-derived IBN in ameliorating colitis and preserving intestinal epithelial barrier function. The effect is mediated through the activation of the peroxisome proliferator-activated receptor γ (PPARγ) signaling pathway, which enhances the expression of tight junction proteins (TJs) in intestinal epithelial cells (IECs).

## Results

### Synergistic effects of ABX and POS on colitis amelioration and gut microbiota restoration

**Fig. 1.**
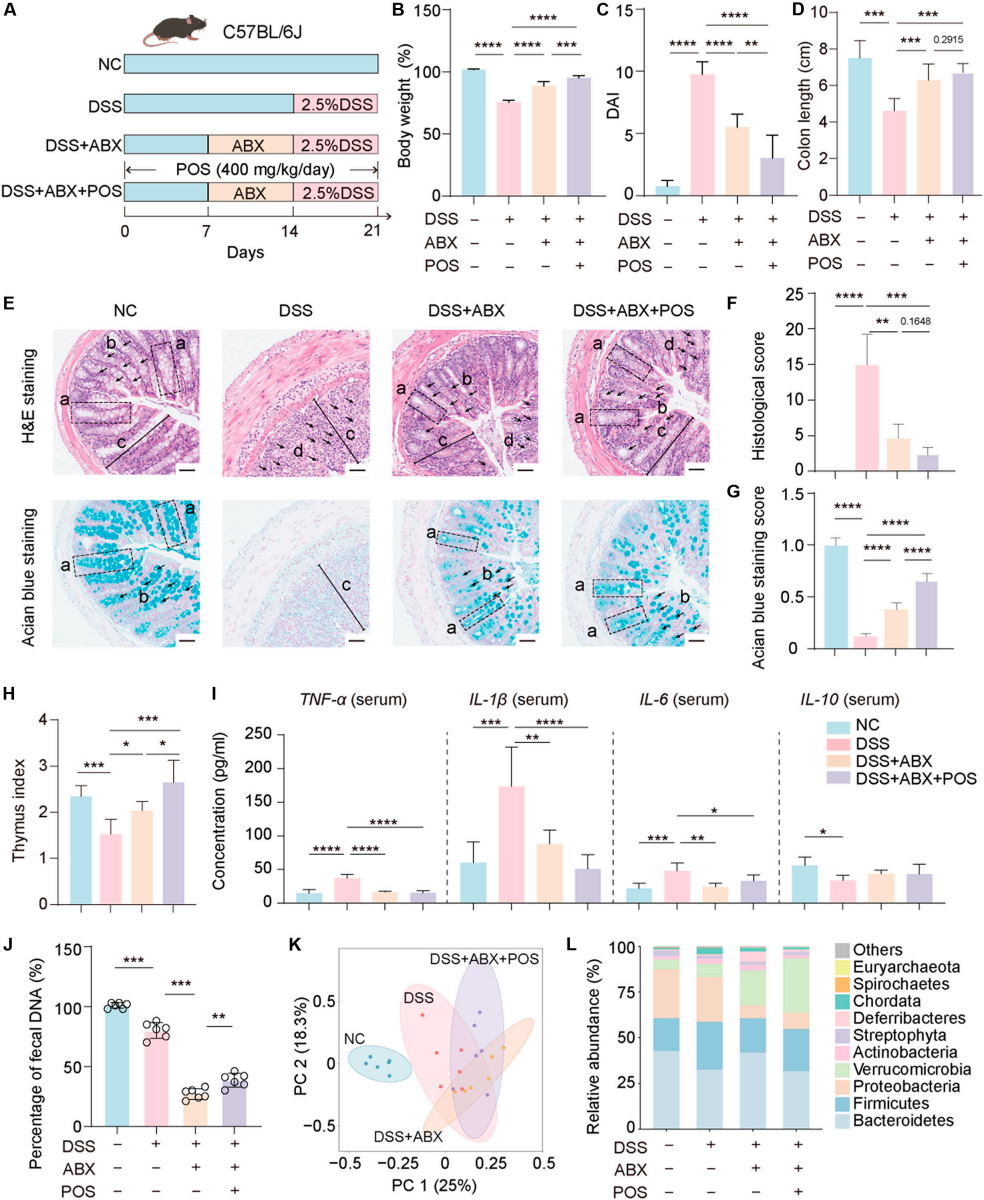
POS and ABX exhibits a synergistic effect in alleviating colitis symptoms. (A) Schematic diagram of experiment design. Body weight change (B), DAI change (C), and colon length (D) for 4 groups (*n* = 8). (E) Histological images (H&E, Alcian blue). Histological score (F) and quantification of Alcian blue staining (G, *n* = 3). (H) Thymus index (*n* = 8). (I) Serum TNF-α, IL-1β, IL-6, and IL-10 in serum (*n* = 8). (J) Total fecal DNA (*n* = 8). (K) PCoA of Bray–Curtis distance. (L) Relative abundance in phylum level. Data are presented as mean ± SD. Compare with the indicated group, *P < 0.05, **P < 0.01, ***P < 0.001, and ****P < 0.0001.

The effectiveness of ABX in alleviating symptoms associated with acute severe colitis and chronic persistent UC has been extensively demonstrated [24]. However, its clinical application is restricted primarily owing to the unavoidable disruption of gut microbial homeostasis. To validate the effectiveness of prebiotic POS supplementation in ABX therapy, we implemented a combined treatment strategy involving both POS and ABX in a mouse colitis model induced by DSS (Fig. 1A). As expected, the general indicators including body weight change, disease activity index (DAI), and colon length exhibited substantial improvement in the damage caused by DSS following treatment with ABX alone or in combination with ABX and POS (ABX+POS) (Fig. 1B to D and Fig. S1A and B). The results of hematoxylin and eosin (H&E) staining as well as Alcian blue staining also demonstrated that DSS-induced colitis led to significant damage to the colon tissue and disruption of the mucous membrane secreted by goblet cells, while ABX alone or ABX+POS treatment could markedly improve these destructions of colon tissue (Fig. 1E to G). Compared to the DSS group, the thymus index of the ABX+POS group showed a significant increase, while the spleen index showed a significant decrease (*P* < 0.05, Fig. 1H and Fig. S1C).

Subsequent analysis revealed that ABX alone and ABX+POS treatment substantially reduce pro-inflammatory factor expression and increase inhibitory inflammatory factor expression in serum and colonic tissues, respectively. However, ABX+POS treatment significantly suppresses the level of interleukin-1β (IL-1β) in serum (*P* < 0.05, Fig. 1I) and tumor necrosis factor-α (TNF-α) in colon tissue (*P* < 0.05, Fig. S1D to G) compared to the ABX group. Treatment with ABX alone and ABX+POS effectively restores the levels of tight junction proteins (ZO-1, Occludin, and Claudin-1) and MUC2 in colon tissues (Fig. S2A and B). Moreover, the ABX+POS group shows more efficacy in enhancing their expression compared to ABX alone. Those results demonstrate that combining ABX and POS provides a synergistic resistance to colitis, ameliorating colonic injury, inflammation, systemic inflammation, and intestinal barrier dysfunction.

To verify the effect of POS on ABX-induced gut microbiota dysbiosis, fecal samples were collected and further subjected to metagenomic sequencing. POS treatment partially restored gut microbiota disrupted by ABX and DSS (Fig. 1J and K and Fig. S3A). ABX+POS treatment markedly reduced Proteobacteria and increased Verrucomicrobia at the phylum level (Fig. 1L and Fig. S3B to E). These findings suggest that the ABX+POS treatment regimen effectively targets both inflammation and microbiota imbalance, offering a potential synergistic approach for managing colitis.

### *P. goldsteinii* as a key bacteria in the colitis pathology and antibiotic resistance

**Fig. 2.**
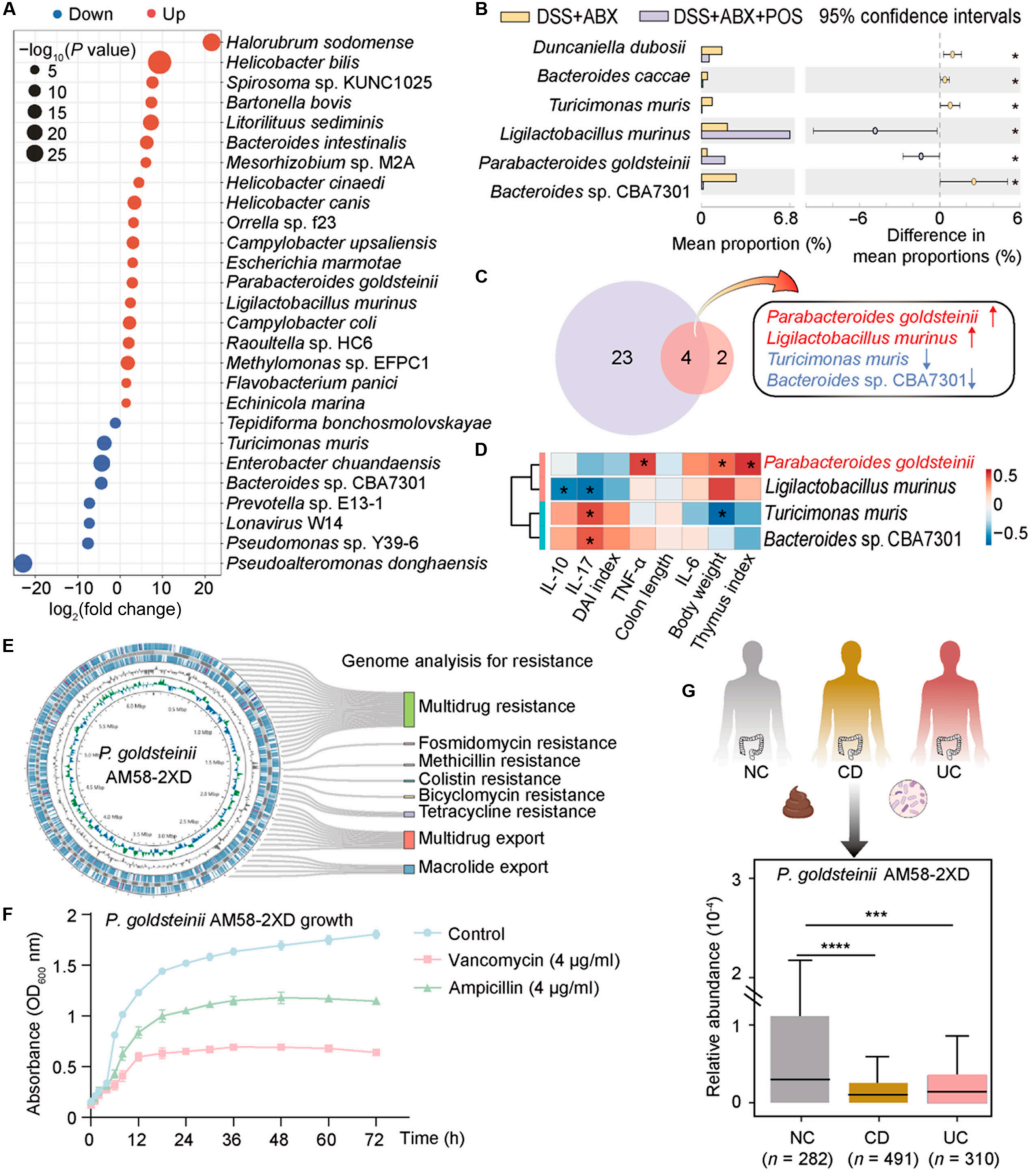
Metagenomic analysis of colitis mice treated with ABX combined with POS. (A) Bubble plot of differential bacteria. (B) Effect size analysis for gut microbiota in ABX and ABX+POS groups. (C) Venn diagram. (D) Spearman correlation heatmap of differentially abundant bacterial taxa with general indicators. (E) Genome of *P. goldsteinii* AM58-2XD and resistance analysis. (F) Growth curves of *P. goldsteinii* AM58-2XD under different antibiotic treatments (*n* = 3). (G) The abundance of *P. goldsteinii* AM58-2XD in CD and UC patients using the human population cohort. Compare with the indicated group, *P < 0.05, ***P < 0.001, and ****P < 0.0001.

In order to elucidate the underlying mechanisms of post-antibiotic microbiome reconstitution facilitated by POS, we conducted a comparative analysis of significant species-level changes in gut microbiota composition (Fig. 2A and Fig. S4A). After ABX+POS treatment, 27 different bacteria were identified (19 increased and 8 decreased) ($\log_{2}|\mathrm{FC}|\;$≥1 and *P*.adjust < 0.05). The antibiotic-induced gut dysbiosis is typically characterized by increases in the abundance of *Enterobacteriaceae* [25,26]. A marked decrease in *Enterobacter chuandaensis* abundance was observed following POS supplementation (*P* < 0.01). Effect size analysis (effect size > 0.2, *P* < 0.05) identified 6 bacteria exhibiting differential abundance between the ABX and ABX+POS groups, including 2 up-regulated strains (Fig. 2B and Fig. S4B to E). Further correlation analysis of 4 selected bacteria and general indexes showed that *P. goldsteinii* was positively correlated with body weight change (*P* < 0.05), thymus index (*P* < 0.05), and TNF-α (*P* < 0.05) (Fig. 2C and D). *P. goldsteinii* may be crucial in modulating the therapeutic effects of synergistic ABX and POS in treating gut microbiota in colitis and alleviating inflammation.

Genomic analysis of *P. goldsteinii* AM58-2XD identified 20 predicted multidrug resistance proteins and 9 multidrug efflux proteins (Fig. 2E). *In vitro* growth experiments showed that *P. goldsteinii* AM58-2XD exhibited a certain degree of tolerance to vancomycin and ampicillin (Fig. 2F). In the population cohort analysis, we observed a significant reduction in the abundance of *P. goldsteinii* AM58-2XD in the intestinal tract of patients with Crohn’s disease (CD, *P* < 0.0001) and UC (*P* < 0.001) (Fig. 2G). The observed enrichment of *P. goldsteinii* under antibiotic exposure may reflect its intrinsic antimicrobial tolerance, potentially linked to resistance-associated genes. Combined with its correlation with beneficial host outcomes, *P. goldsteinii* may play a key role in colitis-related pathology, and therefore, this species was prioritized for functional validation.

### *P. goldsteinii* confers a protective role against colitis related to valine metabolism

**Fig. 3.**
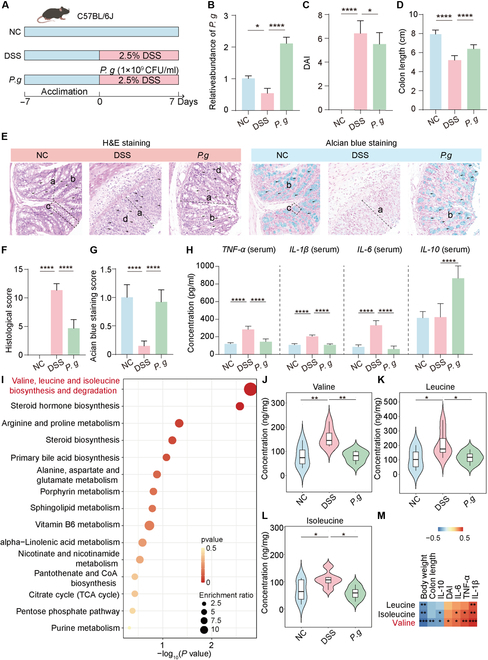
The effect of *P. goldsteinii* AM58-2XD on DSS-induced colitis. (A) Schematic diagram of DSS-induced C57BL/6 mice orally gavaged with *P. goldsteinii*. (B) The relative abundance of *P. goldsteinii* in mice. (C) DAI change on the last day of the experiment. (D) Colon length (*n* = 8). (E) H&E staining images and Alcian blue staining images of colon sections. (F) Histological analysis. (G) The quantitative analysis of Alcian blue staining (*n* = 3). (H) Serum TNF-α, IL-1β, IL-6, and IL-10 in serum (*n* = 8). (I) Differential metabolite enrichment analysis. (J to L) Concentrations of valine, leucine, and isoleucine in fecal samples (*n* = 8). (M) Heatmap of correlation analysis. Compare with the indicated group, *P < 0.05, **P < 0.01, and ****P < 0.0001.

To investigate the effect of *P. goldsteinii* on colitis, we also employed a DSS-induced mouse model and orally administered *P. goldsteinii* AM58-2XD at a dose of 1×10^9^ cfu/100 μl (Fig. 3A). Quantitative real-time polymerase chain reaction (qRT-PCR) was used to analyze the colonization of *P. goldsteinii*. The result showed that the relative abundance of *P. goldsteinii* decreased by 46.5% in colitis mice but increased 3.9-fold after gavage with *P. goldsteinii* AM58-2XD (Fig. 3B), indicating its efficient colonization in the intestinal environment. Meanwhile, *P. goldsteinii* AM58-2XD substantially reduced DSS-induced weight loss (*P* < 0.0001, Fig. S5A and B), DAI elevation (*P* < 0.05, Fig. 3C and Fig. S5C), and colon shortening (*P* < 0.0001, Fig. 3D) in mice. Intestinal morphology analysis demonstrated that *P. goldsteinii* AM58-2XD intervention markedly ameliorated DSS-induced colonic structural damage, with partially restored crypt density (*P* < 0.0001, Fig. 3E and F). Alcian blue staining further revealed that *P. goldsteinii* AM58-2XD enhanced mucin exocytosis and secretion in colonic goblet cells and specifically enhanced the release of mucin granules at the mucosal surface (Fig. 3E and G). In terms of inflammatory regulation, *P. goldsteinii* AM58-2XD markedly reduced levels of pro-inflammatory cytokines TNF-α, IL-1β, and IL-6 while elevating the anti-inflammatory cytokine IL-10 (*P* < 0.001) in both serum and colon tissues of DSS-treated mice. Correspondingly, inflammatory factor gene expression was down-regulated in colonic tissues (Fig. 3H and Fig. S5D to F). Meanwhile, the results of *P. goldsteinii* treatment on mice with noncolitis showed that it had no effect on the body weight and colon length of the mice (Fig. S6A to E), but could enhance the function of the intestinal barrier (Fig. S6F to H). These findings suggest that *P. goldsteinii* AM58-2XD alleviates colitis by reinforcing the mucosal barrier and suppressing inflammatory responses.

To further investigate the role of *P. goldsteinii* in their anticolitis effects in relation to the microbiota, the sterile mouse model, which involves administering ABX to eliminate the intestinal microbiota, was constructed (Fig. S7A). Following ABX treatment, it was observed that *P. goldsteinii* notably alleviated colitis symptoms. Specifically, the body weight of mice treated with *P. goldsteinii* exhibited a notable increase (Fig. S7B), and there was a significant decrease in the DAI (Fig. S7C) and an increase in colon length (Fig. S7D). Histological analyses corroborated these findings, as evidenced by H&E staining and Alcian blue staining, which demonstrated significant improvements following *P. goldsteinii* intervention (Fig. S7E to G). Collectively, these results suggest that *P. goldsteinii* may promote intestinal health and functional recovery through the modulation of the intestinal microbiota.

To identify microbiota-derived metabolites mediating the interaction between *P. goldsteinii* and the host, we conducted untargeted metabolomic profiling of fecal samples. A total of 660 metabolites were detected. Principal coordinates analysis (PCoA) and partial least squares-discriminant analysis revealed distinct clustering of metabolic profiles among the control, DSS-induced colitis, and *P. goldsteinii*-treated groups in both positive and negative ion modes (Fig. S8A to D). Compared to the DSS group, *P. goldsteinii*-treated mice exhibited 524 up-regulated and 100 down-regulated metabolites in the positive ion mode, and 127 up-regulated and 65 down-regulated metabolites in the negative ion mode (Fig. S8E and F). These differentially expressed metabolites were screened based on the criteria of $|\log_{2}\mathrm{FC}|$>1 and *P* < 0.05. Supplementation with *P. goldsteinii* notably reshaped this metabolic landscape, as evidenced by pathway enrichment analysis, which highlighted valine, leucine, and isoleucine biosynthesis and degradation as the most affected pathways (Fig. 3I).

To validate these findings, we performed targeted metabolomics analysis focusing on 22 amino acids (Fig. S9). The concentrations of valine (*P* < 0.05), leucine (*P* < 0.05), and isoleucine (*P* < 0.01) were markedly decreased in *P. goldsteinii*-treated mice compared to DSS-treated controls (Fig. 3J to L). Furthermore, correlation analysis demonstrated that fecal valine levels were positively associated with inflammatory markers and negatively correlated with body weight and colon length (Fig. 3M).

The levels of branched-chain amino acids (BCAAs) in blood samples revealed that in the DSS-induced colitis model, BCAA levels were substantially elevated, while treatment with *P. goldsteinii* led to a marked reduction in these levels (Fig. S10). Taken together, these results indicate that *P. goldsteinii* treatment notably altered BCAA metabolism, with valine, leucine, and isoleucine identified as the key metabolites. Targeted analysis confirmed their reduction (*P* < 0.05), and valine levels were strongly correlated with colitis severity (Fig. 3M), suggesting its central role in *P. goldsteinii*’s protective mechanism.

### Enhanced alleviating effects of valine–IBN metabolism on colitis

**Fig. 4.**
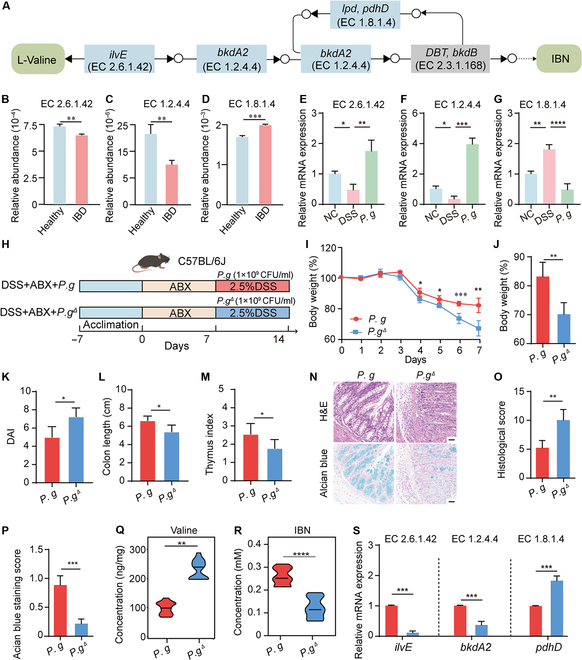
*P. goldsteinii* alleviates colitis related to the valine–IBN metabolic pathway. (A) KEGG maps for valine degradation and IBN synthesis. The relative abundance of EC 2.6.1.42 (B), EC 1.2.4.4 (C), and EC 1.8.1.4 (D) in metagenomic data of the population cohort (healthy: *n* = 429, IBD: *n* = 1,209). The mRNA level of EC 2.6.1.42 (E), EC 1.2.4.4 (F), and EC 1.8.1.4 (G) in fecal samples using qRT-PCR (*n* = 8). (H) Animal experiment design. (I) Body weight change during the experimental period (*n* = 8). (J) The final body weight change. (K) The final DAI. (L) Colon length. (M) Thymus index (*n* = 8). (N) The H&E and Alcian blue staining for colon tissues. (O) The histological score for H&E staining (*n* = 3). (P) Quantitative analysis of Alcian blue staining (*n* = 3). The concentration of valine (Q) and IBN (R) in fecal samples (*n* = 8). (S) The mRNA level of *ilvE*, *bdkA2*, and *pdhD* in fecal samples using qRT-PCR (*n* = 8). Compare with the indicated group, *P < 0.05, **P < 0.01, ***P < 0.001, and ****P < 0.0001. *P.g*, *P. goldsteinii* AM58-2XD; EC 2.6.1.42, branched-chain amino acid aminotransferase (*ilvE*); EC 1.2.4.4, 2-oxoisovalerate dehydrogenase subunit beta (*bkdA2*); EC 1.8.1.4, dihydrolipoyl dehydrogenase (*pdhD*).

The above results suggest a bioactive role for *P. goldsteinii* in modulating valine patterns. Valine participates in various biosynthetic processes within the host, and through the catalysis of specific enzymes, it can be converted into IBN (Fig. 4A). Using the metagenomic analysis of the population cohort, in the samples of inflammatory bowel disease (IBD) patients, it could be observed that the abundance of enzyme genes in the valine–IBN degradation pathway decreased (EC 2.6.1.42: BCAA aminotransferase, EC 1.2.4.4: 2-oxoisovalerate dehydrogenase subunit beta, Fig. 4B and C), while the abundance of enzymes in the synthetic pathway increased (EC 1.8.1.4: dihydrolipoyl dehydrogenase, Fig. 4D). We observed consistent changes in the expression levels of the above enzyme genes in colitis mice treated with *P. goldsteinii* (Fig. 4E to G). Among the various measured SCFAs, interestingly, treatment with *P. goldsteinii* AM58-2XD markedly enhanced the abundance of IBN (*P* < 0.001), which are degradation products of valine (Fig. S11).

Previous studies have indicated that the modulation of gut microbiota-mediated valine degradation can effectively alleviate inflammation [27,28]. However, the precise role of the valine degradation product IBN, particularly in relation to colitis, remains unclear. To further verify the colitis remission effect of the valine–IBN metabolic pathway on *P. goldsteinii*, we constructed the *ilvE* gene knockout *P. goldsteinii* (*P. g*^∆^*^ilvE^*). After using ABX to clear the intestinal flora, the *P. g*^∆^*^ilvE^* lost the alleviating effect on colitis (Fig. 4H). Specifically, it lost its improvement effect on the reduction of body weight (*P* < 0.01, Fig. 4I and J), the increase of DAI (*P* < 0.05, Fig. 4K), the reduction of colon length (*P* < 0.05, Fig. 4L), and the reduction of thymus index (*P* < 0.05, Fig. 4M) in mice with colitis. The improvement effect on the integrity of colonic tissue and the protection of the intestinal barrier has also been lost (Fig. 4N to P). Targeted metabolism was used to detect the contents of valine and IBN in fecal samples. *P. g*^∆^*^ilvE^* could neither reduce the content of valine (Fig. 4Q) nor increase the content of IBN (Fig. 4R). Consistent with the phenotype results, the *ilvE* and *bkdA2* gene in the fecal samples decreased notably and the *pdhD* increased (Fig. 4S), indicating that the pathway of valine degradation into IBN was inhibited. These results indicated that *P. goldsteinii* alleviates colitis progression by relying on the valine–IBN metabolic pathway.

### IBN has a beneficial effect on ameliorating colitis in mice

**Fig. 5.**
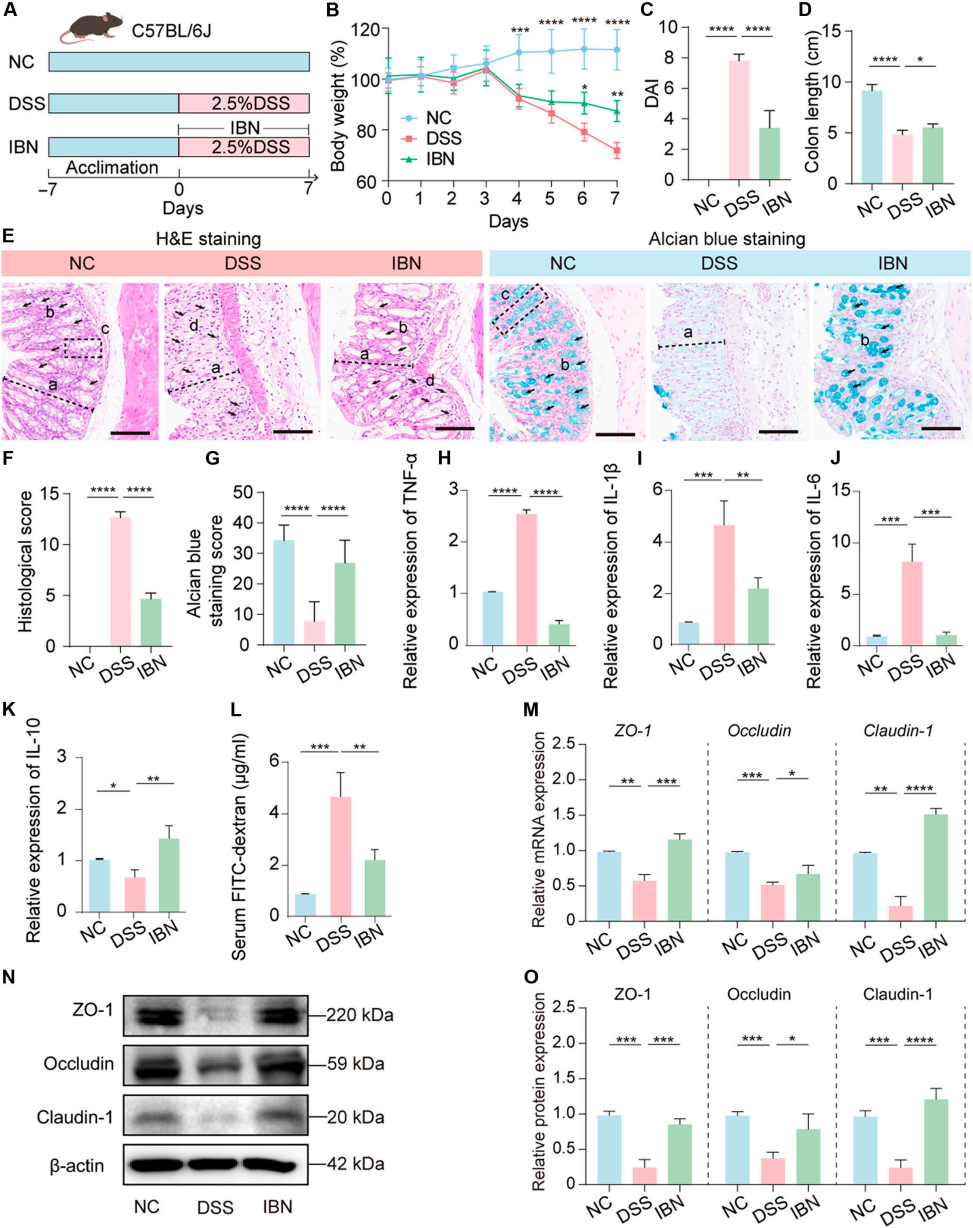
IBN alleviates colitis in DSS-induced mice. (A) Schematic diagram of DSS-induced C57BL/6 mice orally gavaged with IBN. (B) Percentage of body weight change during the experiment period (*n* = 8). (C) The final DAI and (D) colon lengths (*n* = 8). (E) H&E staining and Alcian blue staining images. (F) Histological analysis (*n* = 3). (G) The quantitative analysis of Alcian blue staining (*n* = 3). The TNF-α (H), IL-1β (I), IL-6 (J), and IL-10 (K) in colonic tissues were measured using qRT-PCR (*n* = 8). (L) Quantification of serum FITC-dextran (*n* = 6). (M) The mRNA expression of TJs (ZO-1, Occludin, and Claudin-1) (*n* = 6). (N) The protein level of TJs in colon tissues. (O) The quantitative analysis of protein expression (*n* = 3). IBN, isobutyrate. Compare with the indicated group, *P < 0.05, **P < 0.01, ***P < 0.001, and ****P < 0.0001.

Previous studies investigated the effects of butyrate on colitis in mice [29,30], but the anticolitis effects of IBN and related mechanisms require further clarification. Herein, the role of IBN in ameliorating colitis progression was further investigated by synthesizing glyceryl triisobutyrate in colitis mice (Fig. 5A). We observed that after IBN treatment, the weight loss (Fig. 5B), DAI elevation (Fig. 5C), and colon shortening (Fig. 5D) induced by DSS in mice were substantially reversed, indicating its protective effects at the phenotypic level. To understand the histological basis of these improvements, we examined the structural integrity and mucus layer of the colon. As expected, DSS-induced mice showed disrupted colon structural integrity and loss of crypts (Fig. 5E to G). However, treatment with IBN partially restored the structural integrity of the colon (*P* < 0.0001, Fig. 5E). Furthermore, Alcian blue staining revealed that DSS caused a significant decrease in mucus-producing goblet cells compared to control mice, whereas IBN treatment increased the number of goblet cells and improved the integrity of the colonic mucus layer (Fig. 5E and G). Given that IBN restored the colonic structure, we next investigated its effects on inflammation, a key driver of colitis progression. The results showed that at the molecular level, IBN could down-regulate pro-inflammatory cytokines (TNF-α, IL-1β, and IL-6) while up-regulating the anti-inflammatory cytokine IL-10 (Fig. 5H to K) [31]. An intact intestinal barrier is critical for inflammation. Therefore, to assess the impact of IBN administration on intestinal barrier function, fluorescence spectroscopy of ingested fluorescein isothiocyanate (FITC)-dextran was measured. The results showed that mice IBN treatment revealed an improvement in intestinal permeability compared to DSS-induced colitis mice (*P* < 0.01) (Fig. 5L). To explore the underlying mechanism, we evaluated the expression of tight junction proteins. Both mRNA and protein levels of ZO-1, Occludin, and Claudin-1 were markedly up-regulated in the IBN-treated group compared to the DSS group (Fig. 5M to O). Meanwhile, the results of IBN treatment on mice with noncolitis showed that it had no effect on the body weight and colon length of the mice (Fig. S12A to D), but could enhance the function of the intestinal barrier (Fig. S12E to G). Supplementation of IBN for *P. goldsteinii^ΔiveE^* can still play a role in resisting colitis without affecting colonization (Fig. S13). These results suggest that IBN may combat intestinal inflammation by enhancing the expression of TJs and improving intestinal barrier function, thereby alleviating colitis in mice.

### IBN improves intestinal tight junction through activation of the PPARγ signaling pathway

**Fig. 6.**
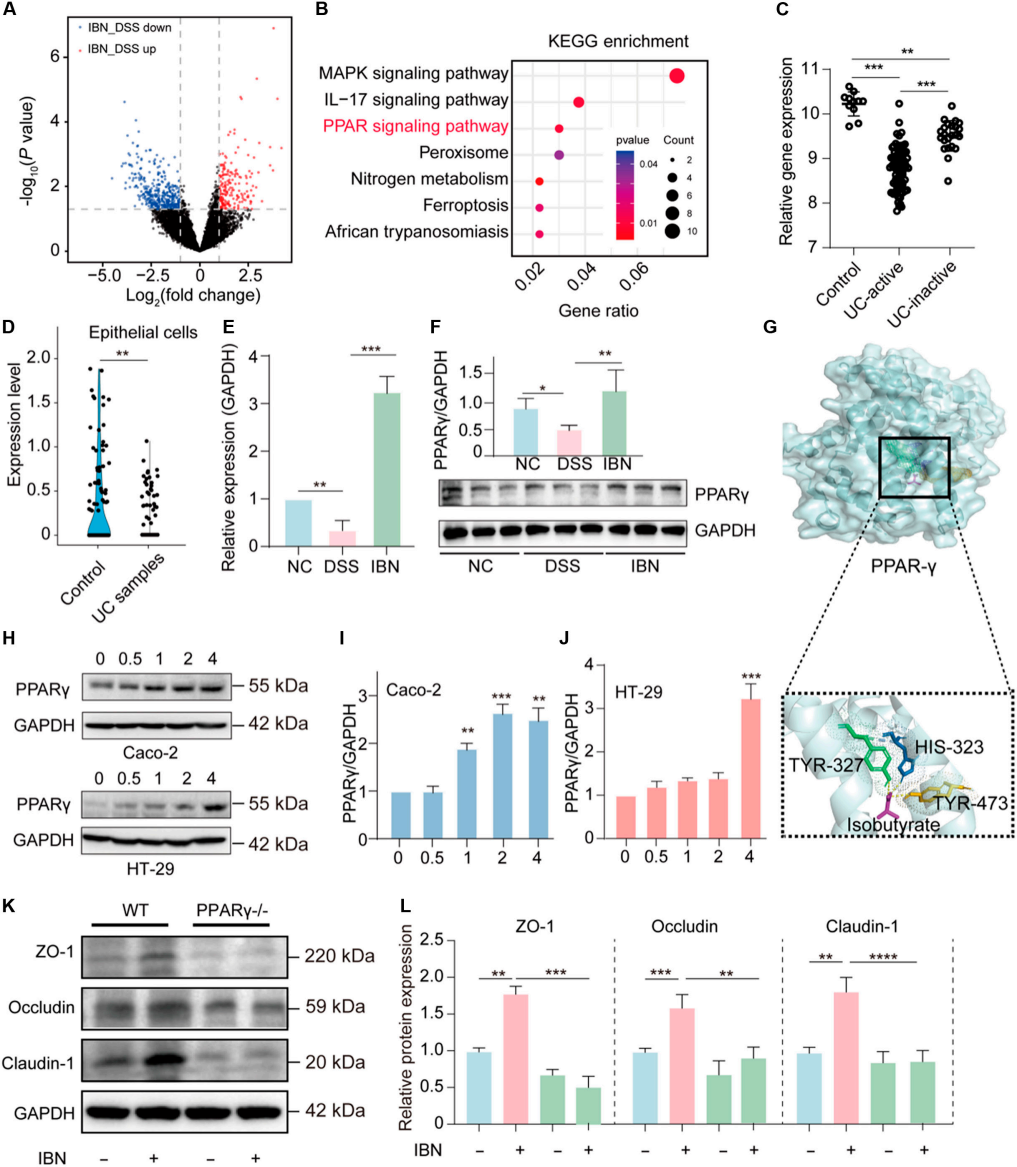
IBN improves intestinal function through activation of the PPARγ signaling pathway. (A) Volcano analysis of gene expression (*n* = 4). (B) KEGG analysis for DEGs. (C) Clinical patient transcriptome analysis of PPARγ gene expression in normal and different periods of UC patients. (D) The expression of PPARγ gene in intestinal epithelial cells was analyzed by single-cell sequencing. (E) The gene expression of PPARγ for colonic samples was examined by qRT-PCR (*n* = 6). (F) The protein level of PPARγ was examined by Western blot and quantitative analysis (*n* = 3). (G) Molecular docking of IBN and PPARγ using the Autodock vina software. The protein level of PPARγ for Caco-2 and HT-29 cells with treatment of IBN (0.5, 1, 2, and 4 mM) was examined by Western blot (H) and quantitative analysis (I and J) (*n* = 3). (K) The protein levels of TJs for Caco-2 and Caco-2^PPARγ−/−^ after IBN treatment. (L) Quantitative analysis of protein expression (*n* = 3). Compare with the indicated group, *P < 0.05, **P < 0.01, ***P < 0.001, and ****P < 0.0001. IBN, isobutyrate.

In order to explore the protective mechanism of IBN on the intestinal tract, transcriptomic analysis was conducted on the colon tissue. Through differential analysis, 685 differentially expressed genes (DEGs, including 199 up-regulated and 486 down-regulated genes) were identified (Fig. 6A). Enrichment analysis of DEGs may function through the mitogen-activated protein kinase (MAPK), IL-17, and PPAR pathways (Fig. 6B). PPARγ is a regulatory factor of nuclear transcription that can be activated by ligands [32]. PPARγ influences host–microbiome interactions by regulating the energy metabolism of colon cells and oxygen supply of the gut microbiome [33]. Transcriptome data from colitis patients (GSE59071) were used to observe PPARγ gene expression (Fig. 6C). Compared with normal colon tissue, PPARγ gene is markedly down-regulated in the colon tissue of patients with active colitis and nonactive colitis (Fig. 6C). In particular, single-cell RNA sequencing analysis (GSE162335) revealed notably reduced PPARγ expression in the colonic epithelium of patients with UC versus normal controls, suggesting impaired PPARγ-mediated transcriptional activity in UC pathogenesis (Fig. 6D). The results indicate that PPARγ plays an important role in the occurrence and development of UC. The elevated mRNA and protein level of PPARγ was further confirmed by qRT-PCR (Fig. 6E) and Western blot (Fig. 6F). It was observed that treatment with IBN in DSS-induced colitis mice increased PPARγ expression at gene and protein levels.

**Fig. 7.**
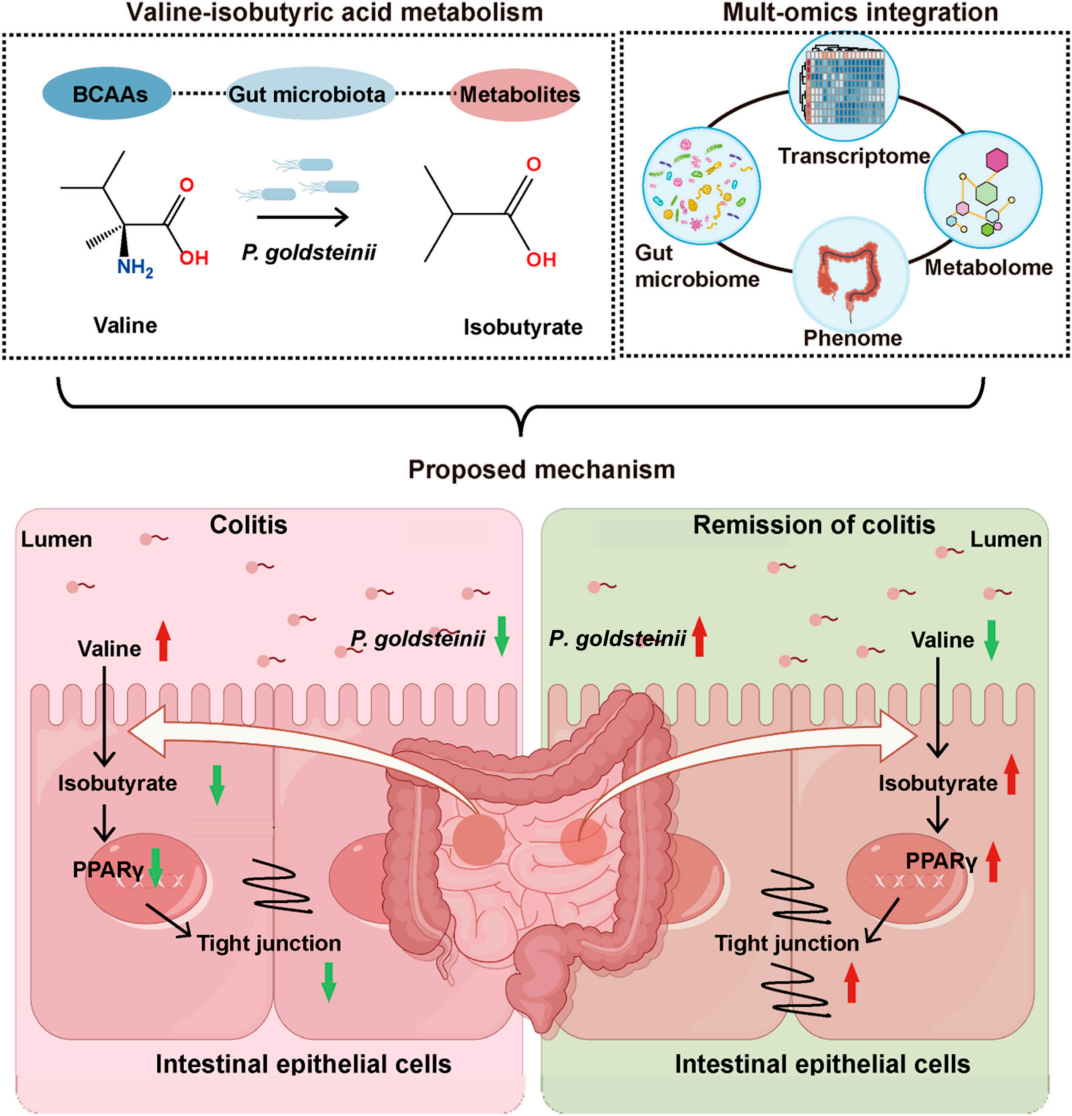
The proposed mechanism of *P. goldsteinii* alleviating UC. The *P. goldsteinii* can complete the metabolism of valine to IBN in the gut. After IBN enters IECs, it activates the PPARγ signal, inhibits the expression TJs in IECs, maintains the function of the intestinal epithelial barrier, and alleviates colitis.

Using the Autodock vina software, the binding site of IBN was located in the LBD domain of PPARγ. Where butyrate binds to tyrosine 327 (TYR-1327), histidine 323 (HIS-323), serine 289 (SER-289), and tyrosine 473 (TYR-473) of PPARγ, IBN binds to tyrosine 327 (TYR-1327), histidine 323 (HIS-323), and tyrosine 473 (TYR-473) of PPARγ (Fig. 6G). Molecular simulations based on energy minimization found that butyrate and IBN have similar binding abilities. Given the close contact of microbial metabolites with the intestinal epithelium, we examined whether gut-derived IBN exerts an anti-inflammatory effect by directly affecting the intestinal epithelium. We evaluated the functional activity of IBN in vitro. As shown in Fig. S14A and B, IBN had no obvious toxicity to human colonic epithelial Caco-2 and HT-29 cells. At the gene level, we also observed that IBN markedly increased the expression of PPARγ and other key genes of the PPARγ signaling pathway (plin1, CD36, Scd1, Slc27a1, Fabp4, and CPT1A) in Caco-2 and HT-29 cells (Fig. S14C to R). Further, IBN can notably enhance the expression of PPARγ at the protein level in Caco-2 and HT-29 cells (Fig. 6H to J). To further explore how IBN alleviates colitis via the PPARγ signaling pathway, an IEC line with stable PPAR knockout (Caco-2^PPARγ−/−^) was constructed. In the case of PPAR knockout, IBN loses the ability to activate TJ expressions (Fig. 6K and L). This indicates that IBN, as a novel endogenous PPARγ agonist, can promote the tight junctions of intestinal epithelium. Furthermore, in vivo experiments have demonstrated that the use of PPARγ inhibitor GW9662 can inhibit the anticolitis effect of IBN (Fig. S15A to D) and substantially reduce the gene expression of the TJs (Fig. S15E to G). Therefore, we hypothesize that IBN produced by the metabolism of valine by *P. goldsteinii* plays a role in resisting colitis by activating the PPARγ signaling pathway, reducing the expression of TJs in IECs, and affecting the integrity of the intestinal epithelial barrier (Fig. 7).

## Discussion

Oral administration of ABX has shown efficacy in inducing remission among patients with acute severe colitis and chronic persistent UC, particularly in cases associated with bacterial infections [34,35]. However, ABX therapy inevitably disrupts intestinal homeostasis, necessitating strategies to regulate gut microbiota dysbiosis [36]. While prebiotic supplementation shows promise in restoring microbiota diversity after ABX, studies on the mechanism of prebiotics in antibiotic therapy are limited. Previous studies indicate that prebiotic pectin demonstrates regulatory effects on gut microbiota and serves as a preventive treatment for antibiotics-induced gut dysbiosis [37]. Our previous study has suggested that enzymatic POS possess the potential to effectively modulate the composition and diversity of gut microbiota [23]. This study further elucidates that POS not only synergize with ABX to enhance anti-inflammatory efficacy but also effectively relieve ABX-induced gut microbiota dysbiosis (Figs. 1 and 2). Our findings propose a potentially effective strategy for ABX-induced gut dysbiosis and adverse effects through the supplementation of prebiotics.

Our results indicated that the ABX+POS treatment specially increased the abundance of *P. goldsteinii* and *Ligilactobacillus murinus* (Fig. 2A to D). Therefore, it is quite possible that *P. goldsteinii* and *L. murinus* could be key species mediating the synergistic antagonistic effects of POS and ABX on colitis. Previous studies have indicated that the *P. goldsteinii* strain exhibits resistance or resilience to antibiotics treatment [22]. Genomic analysis of *P. goldsteinii* AM58-2XD revealed 20 predicted multidrug resistance proteins and 9 multidrug export systems (Fig. 2E), aligning with its documented vancomycin resistance and survival capacity under antibiotic pressure [38]. Specifically, *P. goldsteinii* AM58-2XD not only colonized the intestine in DSS-induced colitis models but also achieved persistent colonization post-ABX-induced microbiota eradication (Fig. S7), a trait likely attributable to its intrinsic antibiotic tolerance. The performance of *P. goldsteinii* AM58-2XD in animal experiments and the results of the population cohort study have provided significant evidence for the role of this strain in the adjuvant treatment of UC. Although multidrug resistance has traditionally been regarded as a pathogenic threat, *P. goldsteinii* AM58-2XD demonstrates the capacity to alleviate colitis and establish colonization under antibiotic treatment conditions, offering new insights into the comprehensive and dialectical understanding of ABX-resistant strains. Moreover, while this study has validated its potential therapeutic effects through animal experiments, vigilance remains necessary regarding the yet undefined risks associated with drug-resistant strains in future clinical applications.

Our multi-omics analyses reveal a critical mechanistic involvement of BCAA metabolism, particularly the valine–IBN pathway. As essential nutrients requiring exogenous intake in mammals due to their inability for endogenous synthesis [39], BCAAs serve as crucial metabolic regulators in immune modulation and intestinal homeostasis maintenance [40]. While prior studies have predominantly focused on the BCAA-mediated activation of the mammalian target of rapamycin (mTOR) pathway [41], the biological significance of their downstream catabolites has yet to be fully elucidated. Herein, we generated a *P. goldsteinii*^ΔilvE^ isogenic mutant through targeted gene knockout and demonstrated a marked attenuation of its anticolitis efficacy compared to the wild-type strain (Fig. 4). This functional impairment provides compelling evidence for the fundamental importance of valine-derived IBN biosynthesis in mediating *P. goldsteinii*’s therapeutic activity. This functional dissection of the valine–IBN axis in *P. goldsteinii* highlights a critical dependency of intestinal homeostasis on microbial IBN biosynthesis.

Thus far, the anticolitis properties of SCFAs, particularly butyrate, have been well-characterized in UC pathogenesis [29,42], and emerging evidence suggests that branched-chain fatty acids (BCFAs) constitute a functionally distinct class of microbial metabolites with underexplored therapeutic potential. Unlike SCFAs primarily derived from dietary fiber fermentation, BCFAs such as IBN originate predominantly from bacterial catabolism associated with valine degradation pathways mediated by microbial enzymes [43]. Strikingly, IBN supplementation elicited a pronounced anticolitis effect, thereby establishing a direct link between this metabolite and intestinal homeostasis. These mechanistic insights could potentially accelerate the development of precision interventions, including engineered probiotics, dietary modulation of valine metabolism, or small-molecule analogs, to effectively harness BCFA-mediated mucosal protection for the management of UC.

Our findings identify IBN as a previously unrecognized microbial-derived activator of the PPARγ signaling pathway, offering mechanistic insight into how *P. goldsteinii* promotes intestinal barrier integrity. Distinct from SCFAs like butyrate, IBN—produced via valine metabolism—engages PPARγ to modulate epithelial transcriptional responses. Its effects on tight junction integrity were abolished in the PPARγ-deficient IEC model, underscoring the pathway’s necessity. These results expand the known immunometabolic roles of BCFAs, positioning IBN as a functionally relevant metabolite that reinforces epithelial defenses through PPARγ-dependent regulation. If we can further verify it using the PPARγ^ΔIEC^ mouse model, it will further enhance the universality of our discovery and the ability to clarify the biological mechanisms involved. While butyrate is well-known for its PPARγ-dependent immunomodulatory and energy-regulatory roles in IECs, our findings demonstrate that IBN also engages this pathway. This highlights an evolutionary convergence in microbial signaling, where structurally distinct molecules target the same nuclear receptor to maintain intestinal homeostasis. Supplementing the data with surface plasmon resonance (SPR)/isothermal titration calorimetry (ITC) binding affinity analyses will offer a more comprehensive understanding of the direct interactions between IBN and PPAR. Notably, characterizing IBN as a PPARγ agonist expands the functional scope of BCFAs beyond amino acid metabolism, showing how microbial ecosystems use diverse strategies to modulate host transcriptional programs. This dual activation of PPARγ by both SCFAs and BCFAs underscores the centrality of this pathway in microbiota-driven epithelial protection. This redundancy may serve as a fail-safe mechanism to preserve IEC barrier integrity during dysbiosis or metabolic stress.

## Conclusion

This study validates the synergistic effect of prebiotics in antibiotic therapy for mouse colitis and highlights the probiotic potential of enriched antibiotic-resistant *P. goldsteinii* through modulation of the valine–IBN axis. Furthermore, our study confirms the therapeutic effect of IBN on mouse colitis and further demonstrates its ability to activate the PPARγ signaling pathway and inhibit pyroptosis in IECs. Those findings have positive implications for advancing the comprehension of antibiotics, probiotics, and prebiotics in their interactions with the host–gut microbiota.

## Materials and Methods

### Materials

DSS was purchased from MP Biomedicals (#160110, Santa Ana, CA, USA). POS was prepared from pectin under the enzymatic hydrolysis of pectinase according to our previous study [44]. *P. goldsteinii* AM58-2XD was isolated and cultured by BGI-Shenzhen (Shenzhen, China). IBN (S976242) was obtained from Macklin Inc. (Shanghai, China). GW9662 (G125880) was purchased from Aladdin Inc. (Shanghai, China). Glyceryl triisobutyrate (purity 99%) was synthesized by Shanghai Shan Tsuen Biological Co., Ltd. (Shanghai, China). Ampicillin (A910962), metronidazole (M813526), vancomycin (V871983), and neomycin (N799581) were purchased from Macklin Inc. (Shanghai, China). The enzyme-linked immunosorbent assay (ELISA) kits were purchased from Thermo Fisher Scientific (Waltham, MA, USA). PPARγ (A11183). The total fecal DNA extraction kit (DP328) was obtained from Tiangen Biochemical Technology Co., Ltd. (Beijing, China). The anti-GAPDH (A19056) was purchased from Abclonal (Wuhan, China). Anti-ZO1 (GB111981), anti-Occludin (GB111401), anti-Claudin-1 (GB112543), and MUC2 (GB120002) were purchased from Servicebio Technology Co., Ltd (Wuhan, China). Cell Counting Kit-8 (CCK-8 kit, #C0038) was purchased from Beyotime (Shanghai, China). FITC-dextran (#FD4, 3-5 kDa) was purchased from Sigma-Aldrich (St. Louis, USA). Twenty kinds of amino acid standards (purity ≥99%) and their internal standards mixture-^13^C,^15^N (767964-1EA) were purchased from Dr. Ehrenstorfer (Augsburg, Germany) and Sigma-Aldrich (Darmstadt, Germany), respectively.

### Animal study

Animal studies were approved by the Ethics Committee of Medical College of Qingdao University (QDU-AEC-2022363, QDU-AEC-2024462, and QDU-AEC -2024460) following the guidelines of the National Institutes of Health (NIH). Six-week-old mice (18–20 g) were kept in a climate-controlled room with a 12-h dark/light cycle and provided unrestricted food. After 1 week of acclimatization, mice were randomly allocated to experimental groups (for experimental groupings, refer to the Supplementary Materials). Throughout the treatment period, daily monitoring included body weight measurements and DAI scoring, which evaluated weight loss, stool characteristics, and hematochezia. Following treatment completion, animals underwent final weight measurement before sample collection under isoflurane anesthesia. Blood and fecal specimens were obtained prior to euthanasia, with spleens excised for index calculation (spleen-to-body weight ratio). Colon tissues were harvested and cryopreserved at −80 °C for subsequent analysis.

### Histological assessment

Tissue specimens were fixed in 10% formalin, paraffin-embedded, and processed for histological evaluation. Sequential staining was performed with H&E followed by Alcian blue after dewaxing. Microscopic examination was conducted using an OLYMPUS microscope (Tokyo, Japan). For objective assessment, H&E-stained sections were scored blindly, whereas Alcian blue-positive areas were quantified through ImageJ-based morphometric analysis.

### Immunohistochemistry and immunofluorescence analysis

Immunohistochemistry and immunofluorescence analyses were conducted following standard procedures. Following antigen retrieval in heated sodium citrate buffer, tissue sections were sequentially treated with 3% hydrogen peroxide and 3% bovine serum albumin at room temperature. Immunostaining was then performed by overnight incubation with primary antibodies (1:500 dilution) at 4 °C, subsequently followed by secondary antibody application. Immunohistochemical sections were then stained with 3,3′-diaminobenzidine (DAB) and hematoxylin. The images of immunohistochemical and immunofluorescence sections were captured using a microscope (OLYMPUS, Tokyo, Japan).

### qRT-PCR and Western blot analysis

The total RNA extraction and reverse transcription procedures were carried out in accordance with the guidelines provided by the RNA extraction kit (#AC0202, Sparkjade, Jinan, Shandong, China). Primers were custom-designed and synthesized by Sangong Biotech (Shanghai, China) as detailed in Table S1. Gene expression levels were determined via the comparative threshold cycle method (2^−ΔΔCT^ method). Tissue samples (100 mg) were processed for protein extraction with radioimmunoprecipitation assay (RIPA) lysis buffer, including protease and phosphatase inhibitors (Beyotime Biotechnology, Shanghai, China). Protein quantification was carried out using the bicinchoninic acid assay. Western blot analysis was performed as previously described [45].

### Enzyme-linked immunosorbent assay

Serum was obtained by centrifuging collected blood samples at 3,000 rpm for 10 min. Following homogenization of colon specimens in normal saline, the supernatant was collected after centrifugation (10,000 rpm, 15 min) for ELISA quantification. Serum and colon tissue proinflammatory cytokines were measured via ELISA per the manufacturer’s protocol.

### Metagenomic sequencing

The methodology for DNA extraction and detection was conducted in accordance with our previous protocol [44]. Metagenomic analysis was carried out using the DNBSEQ T7 sequencing system (BGI, Shenzhen, China), which produced 100-base pair paired-end sequences for all samples. Stringent quality control measures were implemented using fastp (v0.19.4), including criteria such as an average phred score of 20 and a minimum sequence length of 51 base pairs. Reads corresponding to contaminants or non-mouse sequences were first removed from the dataset based on the mouse genome GRCm39. Subsequently, high-quality reads were retained using seqtk (v1.3) for downstream analysis. Taxonomic profiling and species annotation were performed using Kraken2 and Bracken, while functional analysis was conducted by constructing a genome catalog and utilizing MetaWIBELE. Database mapping was carried out using eggnog-mapper in the subsequent analysis. The Huttenhower Lab Galaxy Server was utilized to perform linear discriminant analysis effect size (LEfSe) in order to identify significant features that had significant differences between the 2 groups.

### Transcriptome analysis

After grinding 150 mg of colon tissue, the total RNA was extracted using the RNA extraction kit (#AC0202, Sparkjade) and evaluated for quality using an Agilent 2100 bioanalyzer (Agilent Technologies, Santa Clara, CA, USA). Subsequently, the Agilent 2100 DNA bioanalyzer and Quant-iT PicoGreen dsDNA detection kit were utilized for RNA library construction and assessment of its purity and quality. The quantitative library was sequenced using single-end reads on an Illumina Genome Analyzer (BerryGenomics Co., Ltd., Beijing). Subsequent bioinformatic analysis involved generating volcano plots and identifying DEGs through R software implementation. The criteria for selecting DEGs included an absolute fold change (|FC|) greater than 2 and a *P* value of less than 0.05. The Kyoto Encyclopedia of Genes and Genomes (KEGG) annotation of DEGs was analyzed using the KEGG database available at https://www.genome.jp/kegg/.

### Untargeted metabolomics analysis

The fecal sample (100 mg) underwent homogenization and sonication upon addition to a cold mixture of 1 ml of methanol–acetonitrile–water (2:2:1, v/v), followed by centrifugation for protein removal. Subsequent analysis was conducted using a T3 column (ACQUITY UPLC HSS T3 1.8 μm, 2.1×100 mm, Waters) coupled to a hybrid quadrupole-orbitrap mass spectrometer (QExactive, Thermo Fisher Scientific) via a ultra-high-performance liquid chromatography (UHPLC) system (Ultimate 3000, Thermo Fisher Scientific). For detailed methods, refer to the Supplementary Materials. Each sample was subjected to FullMS and ddMS in positive ion mode, with the mobile phase comprising acetonitrile (A) and 0.1% formic acid (B).

### Human cohort analysis of normal controls and UC patients

The metagenomic sequencing data for the HMP2 [46] and Yunnan (CNP0004022) cohorts were downloaded from https://ibdmdb.org/ and https://db.cngb.org/. In our previous study, Kraken2-build was performed to build a library of human gut bacteria [47]. Kraken2 (Version 2.1.2) and Bracken (Version 1.0.0) were used for annotation at different taxonomic levels.

### The genome analysis of *P. goldsteinii* AM58-2XD and growth assay

We obtained the genome of *P. goldsteinii* AM58-2XD [FASTA (6,397,923 bp; DNA; 264 sequences)] after whole-genome sequencing and assembly [48]. The Prokka was used for genome annotation, and 5,632 CDS, 8 rRNA, 1 tmRNA, and 59 rRNA showed different colors. GC content and GC Skew were used to show sequence composition [49]. *P. goldsteinii* AM58-2XD was routinely cultured in MPYG medium at 37 °C under strict anaerobic conditions. The chamber atmosphere was maintained with a gas mixture of 5% hydrogen, 10% carbon dioxide, and 85% nitrogen, and hydrogen concentration was stabilized at 3.3% using an anaerobic gas infuser. All media and plasticware were pre-reduced in the anaerobic chamber for at least 24 h prior to use. To evaluate bacterial responses to various antibiotics, OD_600_ measurements were conducted as needed using a GENESYS 30 spectrophotometer (Thermo Fisher Scientific) in Balch-type anaerobic tubes (Hungate tubes) under oxygen-free conditions.

### Cell viability and constructing stable knockout cell line

For cell viability test, HT-29 and Caco-2 cells (5 × 10^4^ cells/well) were seeded in a 96-well plate. After 48 h, IBN was added to the 96-well plate at concentrations of 0, 1, 2, and 4 mM. After 24 h of cultivation, cell proliferation was detected using the CCK-8 kit. For cell-related qRT-PCR or Western blot, HT-29 and Caco-2 cells (5 × 10^5^ cells/well) were seeded in a 6-well plate. After 48 h, IBN was added to the 6-well plate at concentrations of 0, 1, 2, and 4 mM. After 24 h of cultivation, the cells were collected, RNA extraction was performed for qRT-PCR, while protein extraction was conducted for Western blotting. To construct stable knockout cell lines, lentiviral vectors containing specific short hairpin RNAs targeting PPARγ were designed and synthesized. HEK293T cells were cotransfected with the lentiviral plasmid, packaging plasmids (pMD2.G and psPAX2), and a calcium phosphate transfection reagent. After 48 h, recombinant lentivirus-containing supernatant was collected, membrane-filtered (0.45 μm), and utilized for cellular infection. Target cells were cultured in complete medium supplemented with lentivirus and polybrene (8 μg/ml) to enhance infection efficiency. Following a 48-h incubation, the medium was replaced, and stable cell lines were selected by continuously culturing in the presence of puromycin, for at least 1–2 weeks. Knockout efficiency was confirmed by Western blotting and qRT-PCR analysis to ensure the successful disruption of the target gene.

### Public database transcriptome and single-cell sequencing analysis

Transcriptome data from colitis patients (GSE59071) including 74 active UC patients, 23 inactive patients, and 11 normal controls were downloaded from the Gene Expression Omnibus (GEO) repository. Single-cell sequencing data (GSE162335) including 5 normal controls and 11 UC patients were also downloaded from the GEO database. Data were analyzed and collated with reference to previous studies [50]. Data were analyzed using the Seurat R package. Low-quality cells and genes were filtered by excluding cells with >5% mitochondrial gene content and retaining only those with >500 and <6,000 detected features. The remaining cells were normalized using the LogNormalize method, and highly variable genes were identified via the “vst” method. The top 25 principal components were used for t-distributed stochastic neighbor embedding (t-SNE) clustering with a resolution of 2. Cell type annotation was performed using the SingleR package and scHCL package.

### Chemical-protein dock

The Autodock vina software was used to simulate the analysis of the interaction between compounds and proteins. The chemical structures of butyrate and IBN were downloaded from PubChem, and the structural coordinate information file of PPARγ Protein was obtained using AlphaFold2 with UniProt ID: P37231. The molecular docking software Autodock Vina was used to perform virtual docking of butyrate and IBN with PPARγ, and then screened according to binding energy. The results were visualized by PyMOL software.

### Measurement of intestinal permeability

Intestinal barrier function was evaluated by measuring plasma levels of FITC-dextran. Four hours before sacrifice on the experimental endpoint, FITC-dextran was administered orally to mice. Following blood collection in heparinized tubes, plasma was separated by centrifugation (12,000×*g*, 10 min, 4 °C). Aliquots (200 μl) of plasma were dispensed into black 96-well plates for fluorescence quantification (excitation/emission: 485/528 nm).

### Statistical analysis

Data analysis was conducted using GraphPad Prism (Version 8.0). For studies involving 2 groups, statistical comparisons between 2 measurements were assessed using unpaired 2-tailed Student’s *t* test. For studies with 3 or 4 groups, one-way analysis of variance was conducted, followed by Tukey’s post-hoc test for group comparisons. The sample size was calculated and analyzed using G*Power software. Spearman’s correlation analysis was also performed. Statistical significance was determined at *P* < 0.05 and denoted by asterisks (*P < 0.05, **P < 0.01, ***P < 0.001, and ****P < 0.0001). Results are presented as mean ± SD.

## Ethical Approval

All the experimental procedures were approved by the Ethics Committee of Medical College of Qingdao University (QDU-AEC-2022363, QDU-AEC-2024462, and QDU-AEC -2024460).

## Data Availability

The authors confirm that the data supporting the findings of this study are available at China National GeneBank DataBase (CNGBdb) with accession no. CNP0004539. All codes used to generate the bioinformatic analyses are available from the lead author upon reasonable request. Any additional information required to reanalyze the data reported in this paper is available from the lead contact upon reasonable request.

## References

[1] Son SU, Suh HJ, Shin KS. Characterization of a novel sulfated-rhamnoglucuronan isolated from Korean seaweed *Ulva pertusa* and its efficacy for treatment of inflammatory bowel disease in mice. Carbohydr Polym. 2024;342: Article 122373. 39048193 10.1016/j.carbpol.2024.122373

[2] Lu J, Shi T, Shi C, Chen F, Yang C, Xie X, Wang Z, Shen H, Xu J, Leong KW, et al. Thiol-disulfide exchange coordinates the release of nitric oxide and dexamethasone for synergistic regulation of intestinal microenvironment in colitis. Research. 2023;6:0204. 37533463 10.34133/research.0204 PMC10393581

[3] Tong L, Hao H, Zhang Z, Lv Y, Liang X, Liu Q, Liu T, Gong P, Zhang L, Cao F, et al. Milk-derived extracellular vesicles alleviate ulcerative colitis by regulating the gut immunity and reshaping the gut microbiota. Theranostics. 2021;11(17):8570–8586. 34373759 10.7150/thno.62046 PMC8344018

[4] Kobayashi T, Siegmund B, le Berre C, Wei SC, Ferrante M, Shen B, Bernstein CN, Danese S, Peyrin-Biroulet L, Hibi T. Ulcerative colitis. Nat Rev Dis Primers. 2020;6(1):74. 32913180 10.1038/s41572-020-0205-x

[5] Du WW, Zhou C, Yang H, Wen S, Chen Y, Chen EX, Yang XH, Li F, Du KY, Yuan H, et al. Aggravated ulcerative colitis via circNlgn-mediated suppression of nuclear actin polymerization. Research. 2024;7:0441. 39183944 10.34133/research.0441 PMC11342054

[6] Montassier E, Valdés-Mas R, Batard E, Zmora N, Dori-Bachash M, Suez J, Elinav E. Probiotics impact the antibiotic resistance gene reservoir along the human GI tract in a person-specific and antibiotic-dependent manner. Nat Microbiol. 2021;6(8):1043–1054. 34226711 10.1038/s41564-021-00920-0 PMC8318886

[7] Sokol H. Probiotics and antibiotics in IBD. Dig Dis. 2014;32(Suppl 1):10–17. 25531348 10.1159/000367820

[8] Suez J, Zmora N, Zilberman-Schapira G, Mor U, Dori-Bachash M, Bashiardes S, Zur M, Regev-Lehavi D, Ben-Zeev Brik R, Federici S, et al. Post-antibiotic gut mucosal microbiome reconstitution is impaired by probiotics and improved by autologous FMT. Cell. 2018;174(6):1406–1423.e16. 30193113 10.1016/j.cell.2018.08.047

[9] Ledder O, Turner D. Antibiotics in IBD: Still a role in the biological era? Inflamm Bowel Dis. 2018;24(8):1676–1688. 29722812 10.1093/ibd/izy067

[10] Wu X, Chen G, Yang L, Lv Z, Wu Y, Liang C, Chen Y, Shao B, Zhang Y, Li H. Comprehensive antibiotic resistome comparison of *Escherichia coli* from irritable bowel syndrome and ulcerative colitis. Curr Res Microb Sci. 2025;8: Article 100398. 40547378 10.1016/j.crmicr.2025.100398 PMC12181972

[11] Jia DJ, Wang QW, Hu YY, He JM, Ge QW, Qi YD, Chen LY, Zhang Y, Fan LN, Lin YF, et al. *Lactobacillus johnsonii* alleviates colitis by TLR1/2-STAT3 mediated CD206(+) macrophages(IL-10) activation. Gut Microbes. 2022;14(1):2145843. 36398889 10.1080/19490976.2022.2145843 PMC9677986

[12] Singh V, Yeoh BS, Walker RE, Xiao X, Saha P, Golonka RM, Cai J, Bretin ACA, Cheng X, Liu Q, et al. Microbiota fermentation-NLRP3 axis shapes the impact of dietary fibres on intestinal inflammation. Gut. 2019;68(10):1801–1812. 30670576 10.1136/gutjnl-2018-316250

[13] Fei Y, Zhang S, Han S, Qiu B, Lu Y, Huang W, Li F, Chen D, Berglund B, Xiao H, et al. The role of dihydroresveratrol in enhancing the synergistic effect of *Ligilactobacillus salivarius* Li01 and resveratrol in ameliorating colitis in mice. Research. 2022;2022:9863845. 35935130 10.34133/2022/9863845 PMC9275091

[14] Fang X, Liu H, du Y, Jiang L, Gao F, Wang Z, Chi Z, Shi B, Zhao X. *Bacillus siamensis* targeted screening from highly colitis-resistant pigs can alleviate ulcerative colitis in mice. Research. 2024;7:0415. 39015206 10.34133/research.0415 PMC11249912

[15] Gros B, Kaplan GG. Ulcerative colitis in adults: A review. JAMA. 2023;330(10):951–965. 37698559 10.1001/jama.2023.15389

[16] Tao C, Zeng W, Zhang Q, Liu G, Wu F, Shen H, Zhang W, Bo H, Shao H. Effects of the prebiotic inulin-type fructans on post-antibiotic reconstitution of the gut microbiome. J Appl Microbiol. 2021;130(3):634–649. 32813896 10.1111/jam.14827

[17] Ekmekciu I, von Klitzing E, Fiebiger U, Neumann C, Bacher P, Scheffold A, Bereswill S, Heimesaat MM. The probiotic compound VSL#3 modulates mucosal, peripheral, and systemic immunity following murine broad-spectrum antibiotic treatment. Front Cell Infect Microbiol. 2017;7:167. 28529928 10.3389/fcimb.2017.00167 PMC5418240

[18] Hempel S, Newberry SJ, Maher AR, Wang Z, Miles JN, Shanman R, Johnsen B, Shekelle PG. Probiotics for the prevention and treatment of antibiotic-associated diarrhea: A systematic review and meta-analysis. JAMA. 2012;307(18):1959–1969. 22570464 10.1001/jama.2012.3507

[19] Chang CJ, Lin TL, Tsai YL, Wu TR, Lai WF, Lu CC, Lai HC. Next generation probiotics in disease amelioration. J Food Drug Anal. 2019;27(3):615–622. 31324278 10.1016/j.jfda.2018.12.011 PMC9307044

[20] Qin F, Zhang M, Yang Q, Wu L, Mao T, Zhou X, Li J, Lai M. Interactions between *Parabacteroides goldsteinii* CCUG 48944 and diet ameliorate colitis in mice via regulating gut bile acid metabolism. iMetaOmics. 2(2): Article e70008. 10.1002/imo2.70008 PMC12806366 41675168

[21] Ezeji JC, Sarikonda DK, Hopperton A, Erkkila HL, Cohen DE, Martinez SP, Cominelli F, Kuwahara T, Dichosa AEK, Good CE, et al. *Parabacteroides distasonis*: Intriguing aerotolerant gut anaerobe with emerging antimicrobial resistance and pathogenic and probiotic roles in human health. Gut Microbes. 2021;13(1):1922241. 34196581 10.1080/19490976.2021.1922241 PMC8253142

[22] Lai HC, Lin TL, Chen TW, Kuo YL, Chang CJ, Wu TR, Shu CC, Tsai YH, Swift S, Lu CC. Gut microbiota modulates COPD pathogenesis: Role of anti-inflammatory *Parabacteroides goldsteinii* lipopolysaccharide. Gut. 2022;71(2):309–321. 33687943 10.1136/gutjnl-2020-322599

[23] Wang H, Liu N, Yang Z, Zhao K, Pang H, Shao K, Zhou Z, Li S, He N. Preventive effect of pectic oligosaccharides on acute colitis model mice: Modulating epithelial barrier, gut microbiota and Treg/Th17 balance. Food Funct. 2022;13(19):9999–10012. 36065954 10.1039/d2fo01448c

[24] Strati F, Pujolassos M, Burrello C, Giuffrè MR, Lattanzi G, Caprioli F, Troisi J, Facciotti F. Antibiotic-associated dysbiosis affects the ability of the gut microbiota to control intestinal inflammation upon fecal microbiota transplantation in experimental colitis models. Microbiome. 2021;9(1):39. 33549144 10.1186/s40168-020-00991-x PMC7868014

[25] Ma J, Song X, Li M, Yu Z, Cheng W, Yu Z, Zhang W, Zhang Y, Shen A, Sun H, et al. Global spread of carbapenem-resistant Enterobacteriaceae: Epidemiological features, resistance mechanisms, detection and therapy. Microbiol Res. 2023;266: Article 127249. 36356348 10.1016/j.micres.2022.127249

[26] Xu R, Feng N, Li Q, Wang H, Li L, Feng X, Su Y, Zhu W. Pectin supplementation accelerates post-antibiotic gut microbiome reconstitution orchestrated with reduced gut redox potential. ISME J. 2024;18(1): Article wrae101. 38857378 10.1093/ismejo/wrae101 PMC11203915

[27] Gart E, van Duyvenvoorde W, Caspers MPM, van Trigt N, Snabel J, Menke A, Keijer J, Salic K, Morrison MC, Kleemann R. Intervention with isoleucine or valine corrects hyperinsulinemia and reduces intrahepatic diacylglycerols, liver steatosis, and inflammation in Ldlr-/-.Leiden mice with manifest obesity-associated NASH. FASEB J. 2022;36(8): Article e22435. 35830259 10.1096/fj.202200111R PMC12166278

[28] Zhou X, Zhang J, Shen J, Cheng B, Bi C, Ma Q. Branched-chain amino acid modulation of lipid metabolism, gluconeogenesis, and inflammation in a finishing pig model: Targeting leucine and valine. Food Funct. 2023;14(22):10119–10134. 37882496 10.1039/d3fo03899h

[29] Wang R, Cao S, Bashir MEH, Hesser LA, Su Y, Hong SMC, Thompson A, Culleen E, Sabados M, Dylla NP, et al. Treatment of peanut allergy and colitis in mice via the intestinal release of butyrate from polymeric micelles. Nat Biomed Eng. 2023;7(1):38–55. 36550307 10.1038/s41551-022-00972-5 PMC9870785

[30] Miranda PM, de Palma G, Serkis V, Lu J, Louis-Auguste MP, McCarville JL, Verdu EF, Collins SM, Bercik P. High salt diet exacerbates colitis in mice by decreasing *Lactobacillus* levels and butyrate production. Microbiome. 2018;6(1):57. 29566748 10.1186/s40168-018-0433-4 PMC5865374

[31] Tang X, Fang M, Cheng R, Niu J, Huang X, Xu K, Wang G, Sun Y, Liao Z, Zhang Z, et al. Transferrin is up-regulated by microbes and acts as a negative regulator of immunity to induce intestinal Immunotolerance. Research. 2024;7:0301. 38274126 10.34133/research.0301 PMC10809841

[32] Hernandez-Quiles M, Broekema MF, Kalkhoven E. PPARgamma in metabolism, immunity, and cancer: Unified and diverse mechanisms of action. Front Endocrinol. 2021;12: Article 624112. 10.3389/fendo.2021.624112 PMC7953066 33716977

[33] Byndloss MX, Olsan EE, Rivera-Chávez F, Tiffany CR, Cevallos SA, Lokken KL, Torres TP, Byndloss AJ, Faber F, Gao Y, et al. Microbiota-activated PPAR-gamma signaling inhibits dysbiotic Enterobacteriaceae expansion. Science. 2017;357(6351):570–575. 28798125 10.1126/science.aam9949 PMC5642957

[34] Luo H, Cao G, Luo C, Tan D, Vong CT, Xu Y, Wang S, Lu H, Wang Y, Jing W. Emerging pharmacotherapy for inflammatory bowel diseases. Pharmacol Res. 2022;178: Article 106146. 35227890 10.1016/j.phrs.2022.106146

[35] Xi W, Li Z, Ren R, Sai XY, Peng L, Yang Y. Effect of antibiotic therapy in patients with ulcerative colitis: A meta-analysis of randomized controlled trials. Scand J Gastroenterol. 2021;56(2):162–170. 33307882 10.1080/00365521.2020.1858958

[36] de Nies L, Kobras CM, Stracy M. Antibiotic-induced collateral damage to the microbiota and associated infections. Nat Rev Microbiol. 2023;21(12):789–804. 37542123 10.1038/s41579-023-00936-9

[37] Dang G, Wang W, Zhong R, Wu W, Chen L, Zhang H. Pectin supplement alleviates gut injury potentially through improving gut microbiota community in piglets. Front Microbiol. 2022;13:1069694. 36569061 10.3389/fmicb.2022.1069694 PMC9780600

[38] Yuan N, Li X, Wang M, Zhang Z, Qiao L, Gao Y, Xu X, Zhi J, Li Y, Li Z, et al. Gut microbiota alteration influences colorectal cancer metastasis to the liver by remodeling the liver immune microenvironment. Gut Liver. 2022;16(4):575–588. 35318288 10.5009/gnl210177 PMC9289841

[39] Yamamoto K, Tsuchisaka A, Yukawa H. Branched-chain amino acids. Adv Biochem Eng Biotechnol. 2017;159:103–128. 27872960 10.1007/10_2016_28

[40] Nie C, He T, Zhang W, Zhang G, Ma X. Branched chain amino acids: Beyond nutrition metabolism. Int J Mol Sci. 2018;19(4):954. 29570613 10.3390/ijms19040954 PMC5979320

[41] Zhang S, Zeng X, Ren M, Mao X, Qiao S. Novel metabolic and physiological functions of branched chain amino acids: A review. J Anim Sci Biotechnol. 2017;8:10. 28127425 10.1186/s40104-016-0139-z PMC5260006

[42] Zhang Y, Ji W, Qin H, Chen Z, Zhou Y, Zhou Z, Wang J, Wang K. Astragalus polysaccharides alleviate DSS-induced ulcerative colitis in mice by restoring SCFA production and regulating Th17/Treg cell homeostasis in a microbiota-dependent manner. Carbohydr Polym. 2025;349(Pt A): Article 122829. 39643403 10.1016/j.carbpol.2024.122829

[43] Rani N, Hazra S, Singh A, Surolia A. Functional annotation of putative fadE9 of *Mycobacterium tuberculosis* as isobutyryl-CoA dehydrogenase involved in valine catabolism. Int J Biol Macromol. 2019;122:45–57. 30316772 10.1016/j.ijbiomac.2018.10.040

[44] Yu S, Wang H, Cui L, Wang J, Zhang Z, Wu Z, Lin X, He N, Zou Y, Li S. Pectic oligosaccharides ameliorate high-fat diet-induced obesity and hepatic steatosis in association with modulating gut microbiota in mice. Food Funct. 2023;14(21):9892–9906. 37853813 10.1039/d3fo02168h

[45] Cui L, He N, Yu S, Pang H, Zhang Z, Wang J, Hao J, Li S. Polysaccharides from *Paecilomyces hepiali* prevent acute colitis in association with modulating gut microbiota and Treg/Th17 immune balance in mice. Molecules. 2023;28(13): Article 4984. 37446646 10.3390/molecules28134984 PMC10343787

[46] Lloyd-Price J, Arze C, Ananthakrishnan AN, Schirmer M, Avila-Pacheco J, Poon TW, Andrews E, Ajami NJ, Bonham KS, Brislawn CJ, et al. Multi-omics of the gut microbial ecosystem in inflammatory bowel diseases. Nature. 2019;569(7758):655–662. 31142855 10.1038/s41586-019-1237-9 PMC6650278

[47] Lin X, Hu T, Chen J, Liang H, Zhou J, Wu Z, Ye C, Jin X, Xu X, Zhang W, et al. The genomic landscape of reference genomes of cultivated human gut bacteria. Nat Commun. 2023;14(1):1663. 36966151 10.1038/s41467-023-37396-x PMC10039858

[48] Zou Y, Xue W, Luo G, Deng Z, Qin P, Guo R, Sun H, Xia Y, Liang S, Dai Y, et al. 1,520 reference genomes from cultivated human gut bacteria enable functional microbiome analyses. Nat Biotechnol. 2019;37(2):179–185. 30718868 10.1038/s41587-018-0008-8 PMC6784896

[49] Grant JR, Enns E, Marinier E, Mandal A, Herman EK, Chen CY, Graham M, van Domselaar G, Stothard P. Proksee: In-depth characterization and visualization of bacterial genomes. Nucleic Acids Res. 2023;51(W1):W484–W492. 37140037 10.1093/nar/gkad326 PMC10320063

[50] Chen K, Shang S, Yu S, Cui L, Li S, He N. Identification and exploration of pharmacological pyroptosis-related biomarkers of ulcerative colitis. Front Immunol. 2022;13: Article 998470. 36311726 10.3389/fimmu.2022.998470 PMC9606687

