# Supplementary material for: Gut Commensal Antibiotic-Resistant Parabacteroides goldsteinii Ameliorates Mouse Colitis through Valine–Isobutyrate Metabolism
Source: Research (Wash D C). 2025 Sep 11;8:0867. doi: 10.34133/research.0867 (PMC12423503; doi:10.34133/research.0867)
Supplement: Supplementary 1 — Supplementary Materials and Methods Tables S1 and S2 Figs. S1 to S15 [file research.0867.f1.docx]

**Gut commensal antibiotic-resistant *Parabacteroides goldsteinii* ameliorates mouse colitis through valine-isobutyrate metabolism**

**Materials and methods**

**Animal grouping**

The following group experiments were conducted after a one-week acclimatization period.

**Experiment 1 (n=8 per group):** (1) NC group: drink water freely throughout the two weeks experiment; (2) DSS group: consume water ad libitum in 1^st^ week, then DSS (2.5%) solution freely in 2^nd^ week; (3) ABX group: mice were treated with the ABX (ampicillin, 200 mg/kg; metronidazole, 200 mg/kg; vancomycin, 100 mg/kg; neomycin, 200 mg/kg) by oral gavage for 5 days to create the pseudo germ-free mice. Freely consume water containing 2.5% DSS in 2^nd^ week; (4) ABX+POS group: mice were treated with the ABX by oral gavage for 5 days. Freely consume water containing 2.5% DSS and POS (400 mg/kg/day) *via* oral gavage (0.1 mL/mouse/day) for in 2^nd^ week.

**Experiment 2 (n=8 per group):** (1) NC group and (2) DSS group were same with experiment 1; (3) *P. goldsteinii* group: freely consume water containing 2.5% DSS and *P. goldsteinii* (1×10^9^ cfu/100 μL) *via* oral gavage (0.1 mL/mouse/day) in 2^nd^ week.

**Experiment 3 (n=8 per group):** (1) NC group: free access to food and water was provided for two weeks. (2) *P. goldsteinii* group: freely consume water and *P. goldsteinii* (1×10^9^ cfu/100 μL) *via* oral gavage (0.1 mL/mouse/day) in 2^nd^ week.

**Experiment 4 (n=8 per group):** (1) NC group: free access to food and water was provided for two weeks. (2) DSS+ABX group: ABX by oral gavage in 1^st^ week and freely drink water containing 2.5% DSS in 2^nd^ week; (3) DSS+ABX+*P.g* group: Underwent identical ABX pretreatment, then received concurrent 2.5% DSS and *P. goldsteinii* (1×10^9^ cfu/100 μL) supplementation *via* oral gavage (0.1 mL/mouse/day) in 2^nd^ week.

**Experiment 5 (n=8 per group):** (1) *P. goldsteinii* group: ABX by oral gavage in 1^st^ week and freely drink water containing 2.5% DSS and *P. goldsteinii* (1×10^9^ cfu/100 μL) in 2^nd^ week. (2) *P. goldsteinii^∆ilvE^* mutation (*P. g*^∆^) group: ABX by oral gavage in 1^st^ week and freely drink water containing 2.5% DSS and *P. g*^∆^ (1×10^9^ cfu/100 μL) in 2^nd^ week.

**Experiment 6 (n=8 per group):** (1) NC group and (2) DSS group were same with experiment 1; (3) IBN group: freely drink water containing 2.5% DSS and glyceryl triisobutyrate (400 mg/kg/day) *via* oral gavage (0.1 mL/mouse/day) in 2^nd^ week.

**Experiment 7 (n=8 per group):** (1) NC group: free access to food and water was provided for two weeks. (2) IBN group: freely consume water and glyceryl triisobutyrate (400 mg/kg/day) *via* oral gavage (0.1 mL/mouse/day) in 2^nd^ week.

**Experiment 8 (n=8 per group):** (1) DSS+ABX+*P.g* group: underwent identical ABX pretreatment, then received concurrent 2.5% DSS and *P. goldsteinii* (1×10^9^ cfu/100 μL) supplementation *via* oral gavage (0.1 mL/mouse/day) for in 2^nd^ week. (2) DSS+ABX+*P.g^∆^* group: underwent identical ABX pretreatment, then received concurrent 2.5% DSS and *P. g^∆^* (1×10^9^ cfu/100 μL) *via* oral gavage (0.1 mL/mouse/day) for in 2^nd^ week. (3) DSS+ABX+*P. g^∆^*+IBN group: underwent identical ABX pretreatment, then received concurrent 2.5% DSS, and *P. goldsteinii* (*P. g^∆^*) and glyceryl triisobutyrate (400 mg/kg/day) *via* oral gavage (0.1 mL/mouse/day) for in 2^nd^ week.

**Experiment 9 (n=8 per group):** (1) IBN group: freely drink water containing 2.5% DSS and glyceryl triisobutyrate (400 mg/kg/day) *via* oral gavage (0.1 mL/mouse/day) in 2^nd^ week. (2) IBN + GW9662 group: freely drink water containing 2.5% DSS, and glyceryl triisobutyrate (400 mg/kg/day) *via* oral gavage (0.1 mL/mouse/day) and intraperitoneal injection of GW9662 (1mg/kg/day) in 2^nd^ week.

**2. Metabolomics analysis**

**Experiment 1: Non-target metabolomics analyses (n=8 per group)**

Each fecal sample were added 1 mL of a methanol/acetonitrile/water (2:2:1) solvent and homogenized, vortexed, and sonicated for 10 minutes after freezing and thawing in liquid nitrogen. To remove proteins, samples were kept at -20°C for 1 hour, then centrifuged at 13,000 rpm for 15 minutes at 4°C. 1 mL of the supernatant was transferred to new tubes and dried under vacuum. The residue was redissolved in 150 µL of acetonitrile/water (1:1), sonicated for 10 minutes at 4°C, and centrifuged again at 13,000 rpm for 15 minutes at 4°C. LC-MS/MS analysis was conducted using a Thermo Scientific UHPLC system with a T3 column, coupled to a QExactive hybrid quadrupole-Orbitrap mass spectrometer. The mobile phase consisted of (A) acetonitrile and (B) either 0.1% aqueous formic acid (positive mode) or 5 mM NH_4_OAc in water (negative mode). A gradient elution program was employed: 1% B (0-1 min), linear increase to 99% B (1-13 min), hold at 99% B (13-16 min), return to initial conditions (16.5 min), and re-equilibration at 1% B (16.5-20 min), with a constant flow rate of 0.25 mL/min. Injection volumes were 3 μL (positive) and 5 μL (negative). H-ESI source parameters included: spray voltage ±3.5/2.8 kV, capillary and probe temperatures of 320°C and 300°C respectively, sheath/auxiliary gas flows of 40/10, and S-Lens RF level at 50. Full-scan MS data were acquired at 140,000 resolution (m/z 70-1000) with 17,500 fwhm resolution for MS/MS scans. Eight precursor ions were fragmented at 20, 35, or 50 eV collision energy. The AGC target was 1×10^5^, with a maximum injection time of 50 ms. Sample was analyzed in positive and negative ion modes using Full-MS and dd-MS mode.

**Experiment 2: Target metabolomics analyses for amino acids (n=8 per group)**

Each sample was homogenized in 1.5 mL of 0.1% formic acid using a tissue grinder, followed by two freeze-thaw cycles in liquid nitrogen. After vortexing in a 75°C water bath for 15 min and ultrasonication (40 kHz, 10 min), the mixture was centrifuged (10,000 rpm, 5 min) for amino acid analysis. A 100 µL aliquot of the supernatant was combined with 50 µL of internal standard, diluted to 1 mL with 0.1% formic acid, and filtered (0.22 µm PES membrane). Subsequently, 2 µL of the processed sample was loaded onto an Imtakt Intrada Amino Acid column (50 × 3 mm, 3 µm) maintained at 35°C, using an LC-20A system (Shimadzu, Japan) coupled to a Sciex 5500 Qtrap mass spectrometer (USA). The flow rate was 0.5 mL/min. Solvent A was 100 mM ammonium formate in water, and solvent B was 95% acetonitrile in water with 0.3% formic acid. The gradient increased from 12% to 100% A in 7 minutes, held at 90% A for 6 minutes, then returned to initial conditions and held for 3 minutes.

**Experiment 3: Target metabolomics analyses for SCFAs (n=8 per group)**

Fecal samples (300 mg) were utilized for the extraction of SCFAs by Shanghai Luming Biological Technology Co., Ltd (Shanghai, China). Briefly, the samples underwent centrifugation, and the supernatant was collected following extraction with a 50% acetonitrile aqueous solution (v/v). Subsequently, a derivatization treatment was performed using 200 mM 3-NPH prepared in a 50% acetonitrile aqueous solution (v/v) and 120 mM EDC-6% pyridine prepared in a 50% acetonitrile aqueous solution (v/v). The derivatized samples were then introduced into a gas-phase vial for mass spectrometric analysis. Quantification of SCFAs was performed by gas chromatography-mass spectrometry (GC-MS).

**Table S1.** The primers sequence for qRT-PCR.

|  | Forward (5’-3’) | Reverse (5’-3’) |
| --- | --- | --- |
| Mouse-TNF-α | TATGGCCCAGACCCTCACA | GGAGTAGACAAGGTACAACCCATC |
| Mouse-IL-10 | GACTGGCATGAGGATCAGCA | CCGCAGCTCTAGGAGCATGT |
| Mouse-IL-1β | GAGCACCTTCTTTTCCTTCATCTT | TCACACACCAGCAGGTTATCATC |
| Mouse-IL-6 | TAGTCCTTCCTACCCCAATTTCC | TTGGTCCTTAGCCACTCCTTC |
| Mouse-ZO-1 | CTGGTGAAGTCTCGGAAAAATG | CATCTCTTGCTGCCAAACTATC |
| Mouse-Occlaudin | TGCTTCATCGCTTCCTTAGTAA | GGGTTCACTCCCATTATGTACA |
| Mouse-Claudin-1 | AGATACAGTGCAAAGTCTTCGA | CAGGATGCCAATTACCATCAAG |
| Mouse-MUC2 | CCTGAAGACTGTCGTGCTGT | GGGTAGGGTCACCTCCATCT |
| Mouse-PPARG | TCCCGCTGACCAAAGCAAAGGC | CCACGGAGCGAAACTGACACCC |
| Mouse-GAPDH | GGTTGTCTCCTGCGACTTCA | TGGTCCAGGGTTTCTTACTCC |
| Human-FABP4 | TGGGAACCTGGAAGCTTGTCTC | GAATTCCACGCCCAGTTTGA |
| Human-SCD1 | ATGCTCCAAGAGATCTCCAGTTCT | ATGCTCCAAGAGATCTCCAGTTCT |
| Human-CD36 | GGAACTGTGGGCTCATTGC | CATGAGAATGCCTCCAAACAC |
| Human-HMGCS2 | CCGTATGGGCTTCTGTTCAG | AGCTTTGTGCGTTCCATCAG |
| Human-PPARG | GGAAGACCACTCGCATTCCTT | GTAATCAGCAACCATTGGGTCA |
| Human-CPT1A | CTCAGTGGGAGCGACTCTTCA | GGCCTCTGTGGTACACGACAA |
| Huma-SLC27A1 | TGCCGAGAGTGGAACACAC | AAAAGCAGCTGGACCCTACA |
| Human-PLIN1 | ACCTGCCTTACATGGCTTGTT | CCATGGTCTGCACGGTGTAT |
| Human-GAPDH | TGCACCACCAACTGCTTAGC | GGCATGGACTGTGGTCATGAG |
| EU | CGGCAACGAGCGCAACCC | CCATTGTAGCACGTGTGTAGCC |
| Total bacteria 16s rRNA (BAC1) | ACTCCTACGGGAGGCAGCAGT | GTATTACCGCGGCTGCTGGCAC |
| Total bacteria 16s rRNA (BAC2) | AAACTCAAAKGAATTGACGG | CTCACRRCACGAGCTGAC |
| P. goldsteinii | GAATAAAGTGAGCAACGTGTT | AACTTTCACCGCTGACTTAATTA |
| ilvE_1 | TTTTGTTCCTCCCTATGA | GATGTAACTGTTTCCACGA |
| BKDA2 | AGATTTCCTGTTCCCTTA | CGATGTTCCATTCTGTTT |
| lpd | CGGTGCGATGGATAAGGAGA | CGTTAGGGTGGGAGGTT |
| pdhD | TCTCTTTGCTTGCCCATA | CGTTTCCTTGCTCGTTTT |
| ilvE_2 | TGTTTGTTGCTCCGATTCCTA | TCGCTCCACACTTAATTCTTG |

**Table S2.** Absolute (OTUs) for each group.

| Group | Absolute (OTUs) |
| --- | --- |
| NC | 18.64 ± 7.8 |
| DSS | 30.18 ± 29.72 |
| ABX | 15.33 ± 9.59 |
| ABX+POS | 30.95 ± 31.87 |


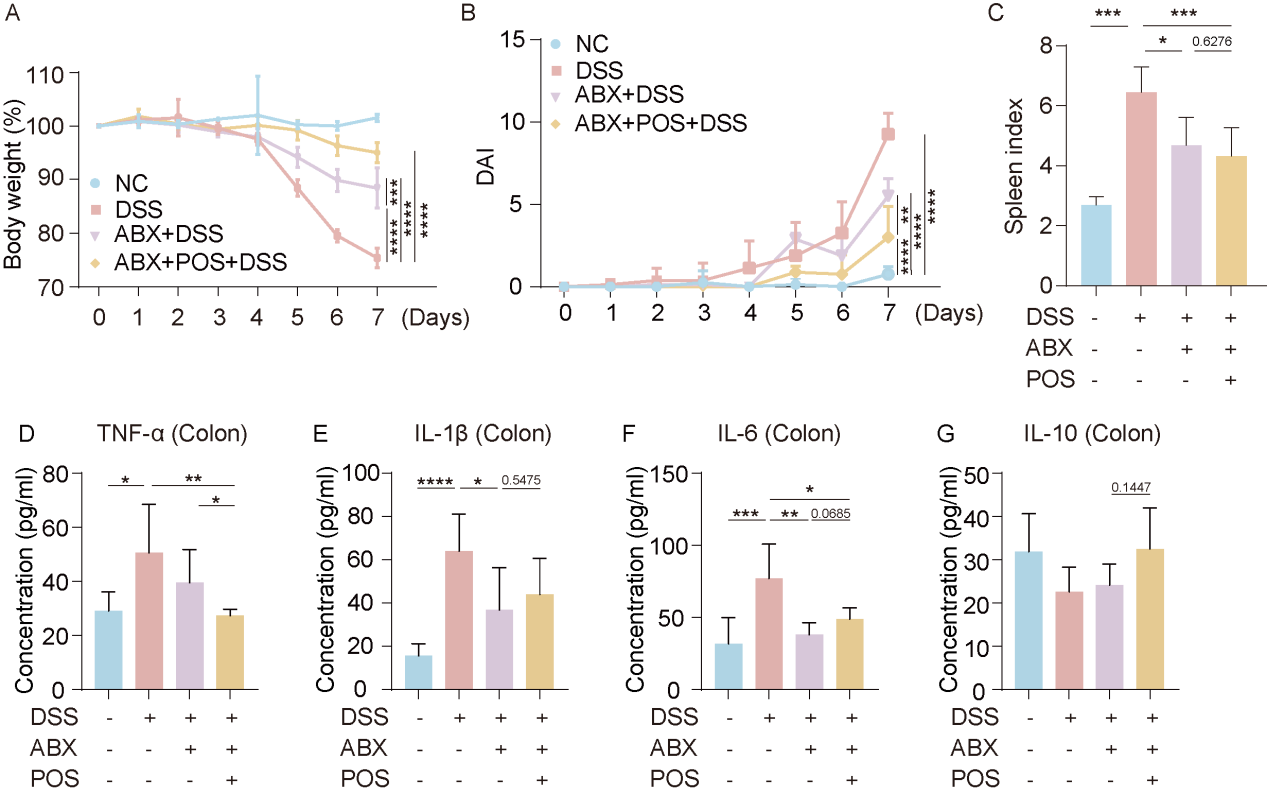


**Figure S1.** ABX combined with POS has synergistic resistance to colitis. The body weight change (A) and DAI index change (B) for four groups during experimental period. (C) Spleen index. The TNFα (D), IL-1β (E), IL-6 (F) and IL-10 (G) in colonic tissues were measure using ELISA (n=8). Data are presented as mean±SD. Compare with indicated group, *P<0.05, **P<0.01, ***P<0.001, ****P<0.0001.


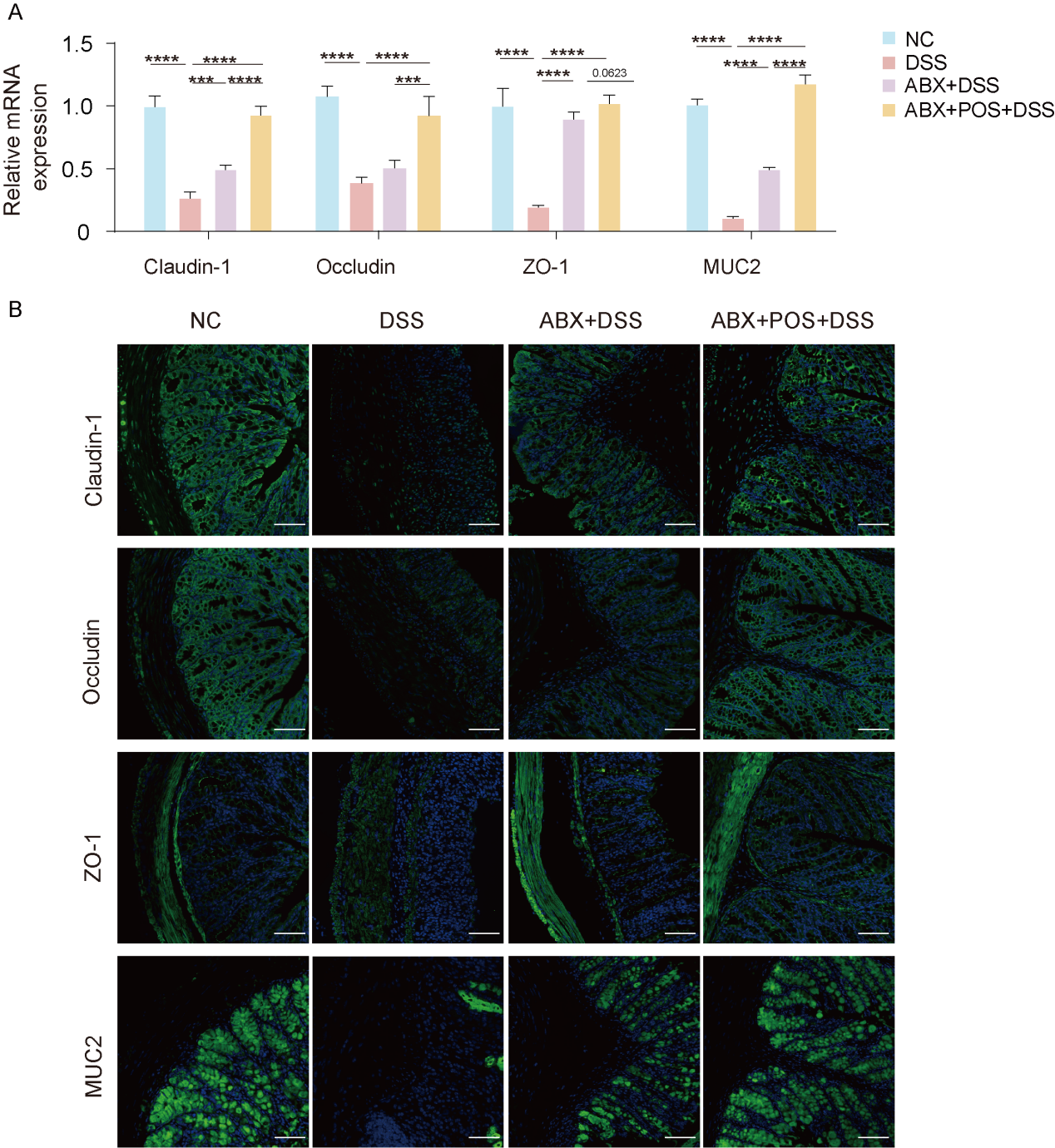


**Figure S2.** Protective effect of ABX combined with POS treatment on intestinal barrier. Tight junction proteins (ZO-1, occludin and claudin-1) and MUC2 in colon tissues were detected using qRT-PCR (A) and immunofluorescence (B), Data were presented as mean±SD (n=3). Compare with indicated group, ***P<0.001, ****P<0.0001.

**
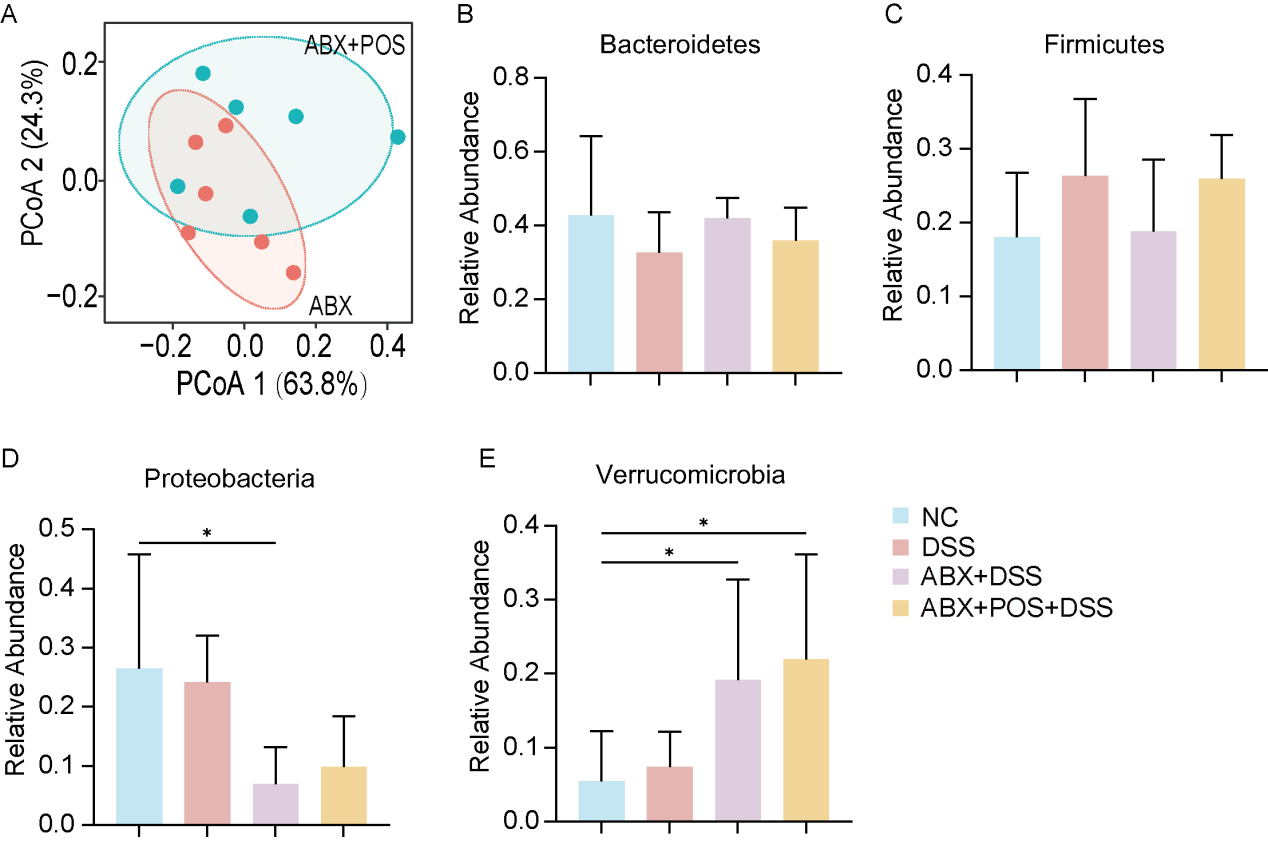
Figure S3.** Regulation effect of ABX combined with POS treatment on gut microbiota. (A) PCoA analysis for ABX and ABX+POS groups. The abundance of gut microbiota in Bacteroidetes (B), Firmicutes (C), Proteobacteria (D) and Verrucomicrobia (E) at the phylum level. Data were presented as mean±SD (n=6). Compare with indicated group, *P<0.05.


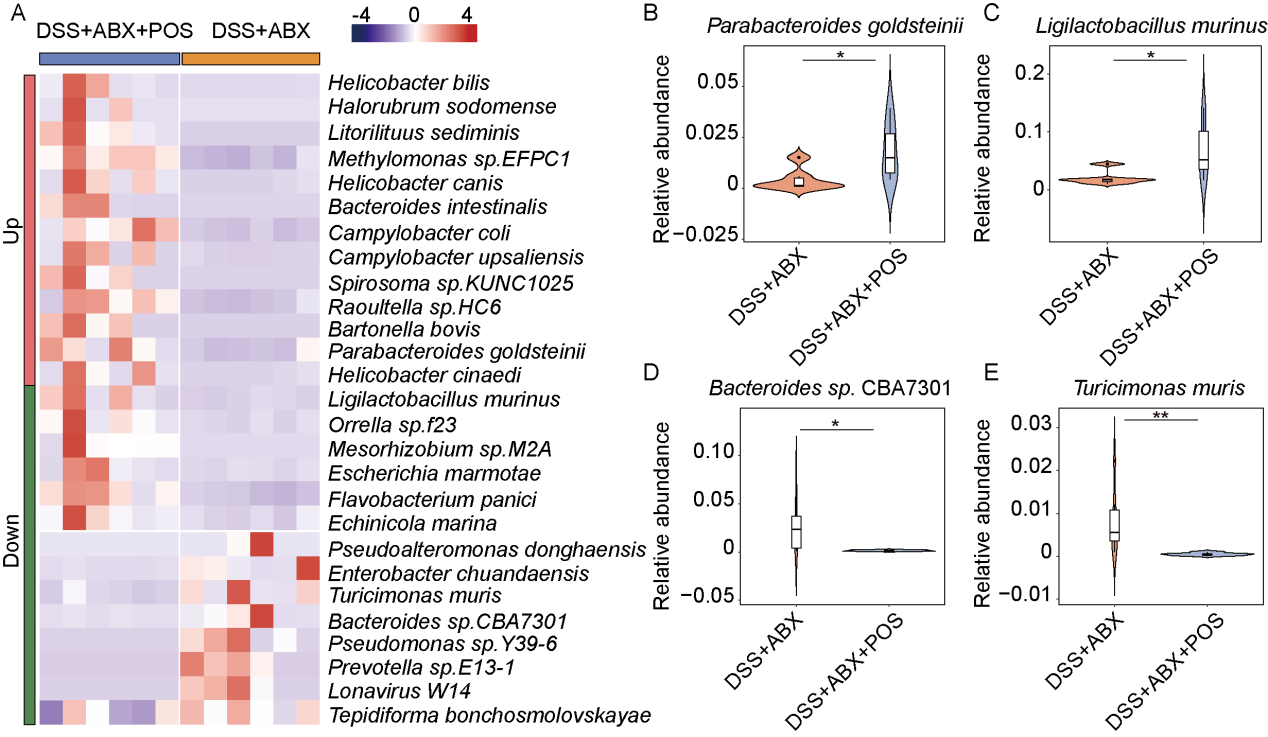


**Figure S4.** Analysis of differential bacterial for ABX and ABX+POS groups in species level. (A) heatmap for differential bacteria. (B) The relative abundance for *Parabacteroides goldsteinii* (B), *Ligilactobacillus murinus* (C), *Bacteroides* sp. CBA7301 (D) and *Turicimonas muris* (E) for ABX and ABX+POS groups. Data were presented as mean±SD (n=6). Compare with indicated group, *P<0.05 and **P<0.01.


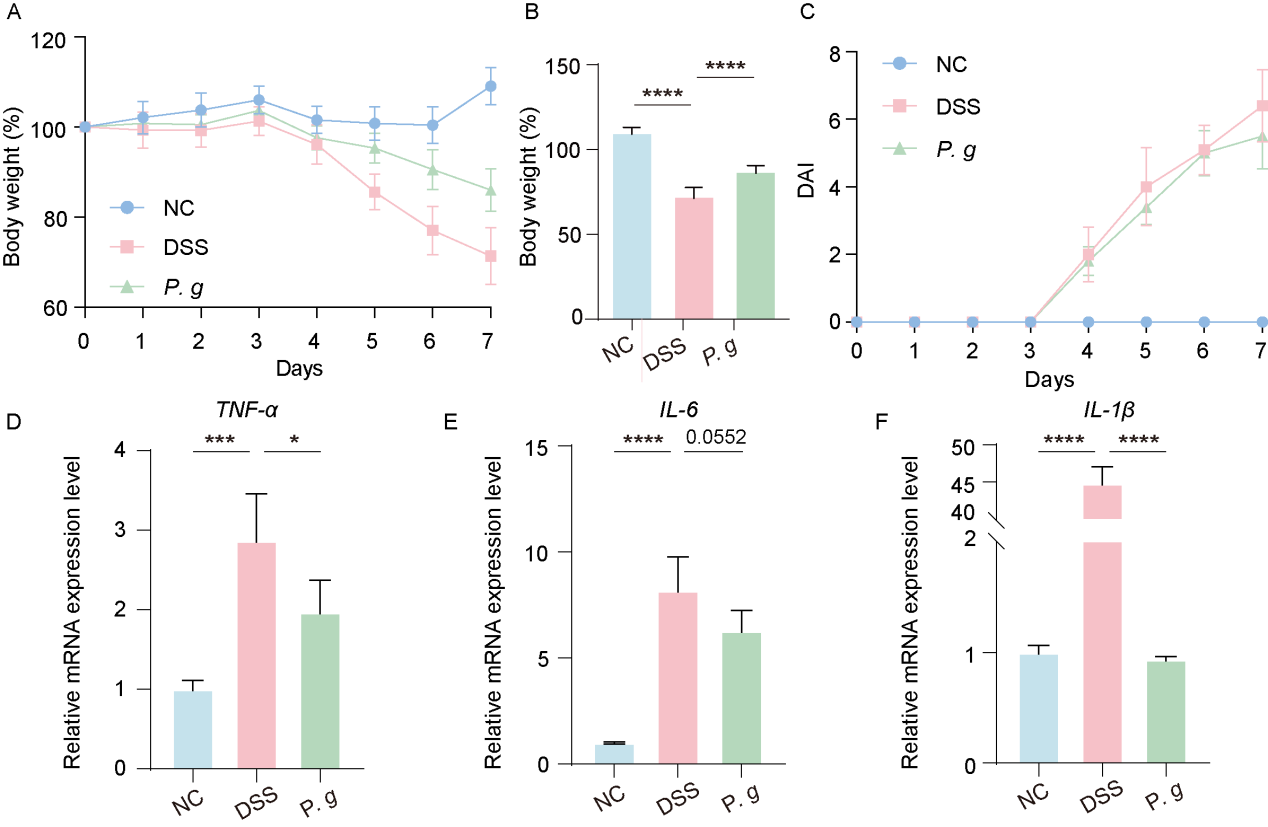


**Figure S5.** Effect of *P. goldsteinii* on DSS induced colitis and the expression of inflammatory factors in colon. (A) Percentage of body weight change during experiment period. (B) The final body weight change. (C) DAI index change during experiment period. The TNF-α (D), IL-6 (E) and IL-1β (F) in colonic tissues were measure using qRT-PCR. Data are presented as mean±SD (n=8). Compare with indicated group, *P<0.05, ***P<0.001, ****P<0.0001.


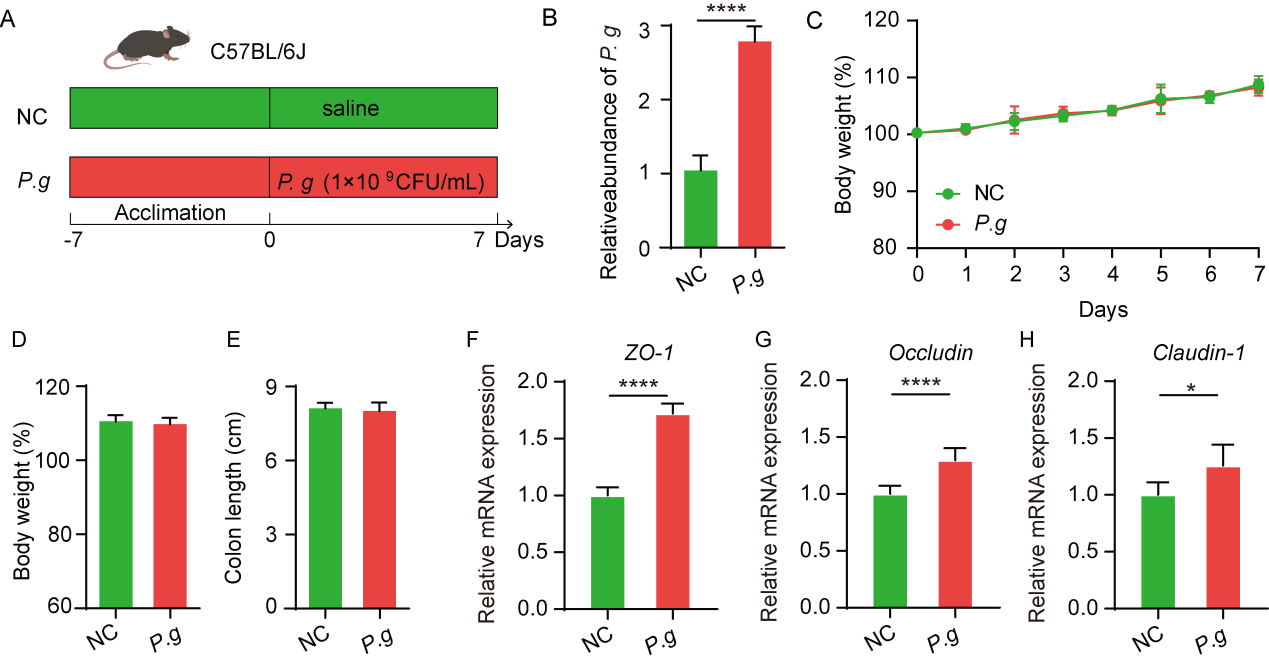


**Figure S6.** Effect of *P. goldsteinii* on normal mice. (A) Experimental design. (B) The abundance of *P. goldsteinii.* (C) Percentage of body weight change during experiment period. (D) The final body weight change. (E) Colon length. The *ZO-1* (F), *Occludin* (G) and *Claudin-1* (H) in colonic tissues were measure using qRT-PCR (n=6). Data are presented as mean±SD. Compare with indicated group, *P<0.05, ****P<0.0001.


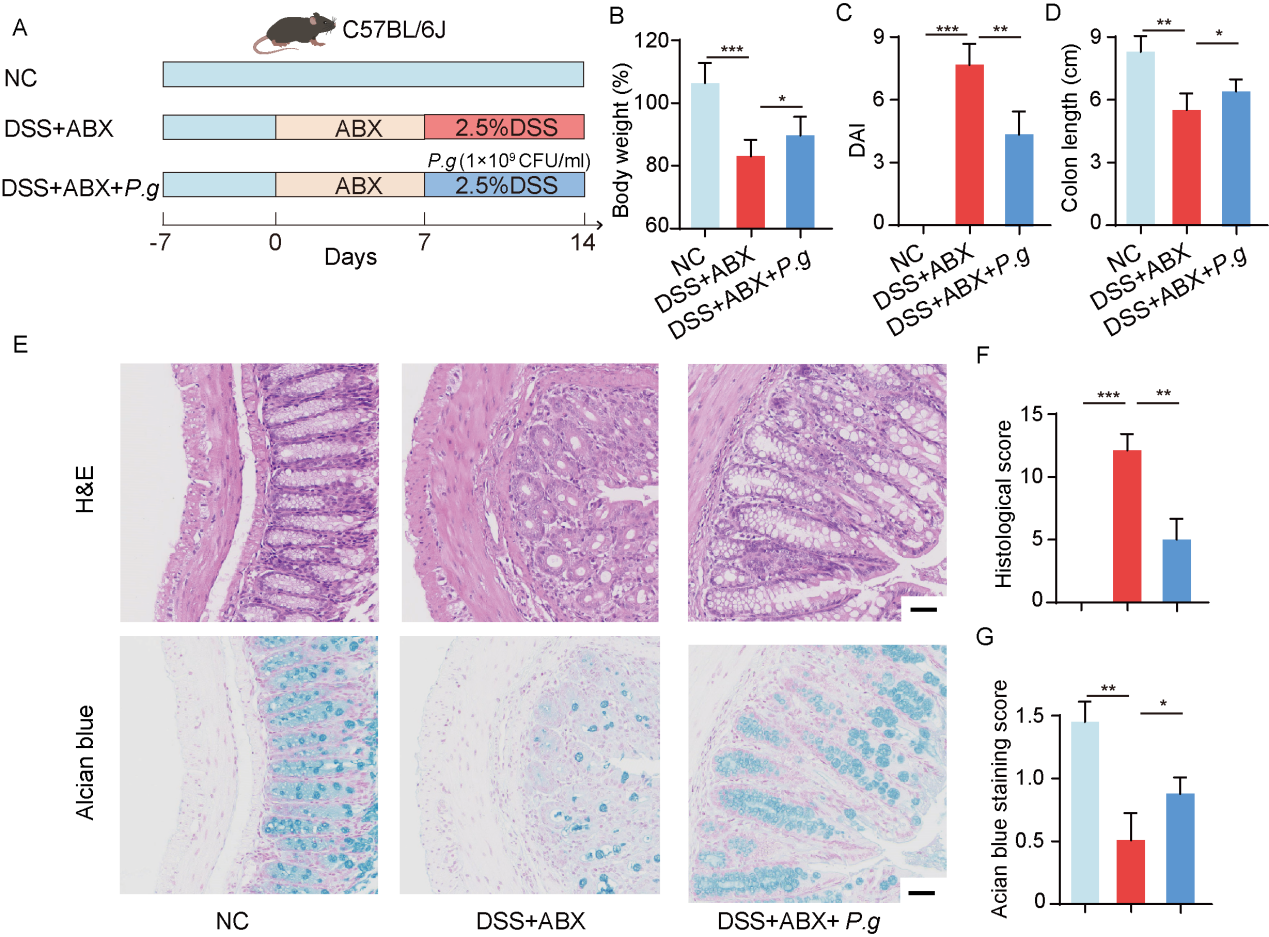


**Figure S7.** The anti colitis effect of *P. goldsteinii* using ABX treated simulated sterile mice. (A) Animal Experiment Design. (B) The percentage of final body weight change. (C) The final DAI. (D) The colon length. N=8. (E) The H&E staining and alcian blue staining. (F) The histological score for H&E staining. (G) Quantitative analysis of alcian blue staining (n=3). Data are presented as mean±SD. Compare with indicated group, *P<0.05, ***P<0.001, ****P<0.0001.


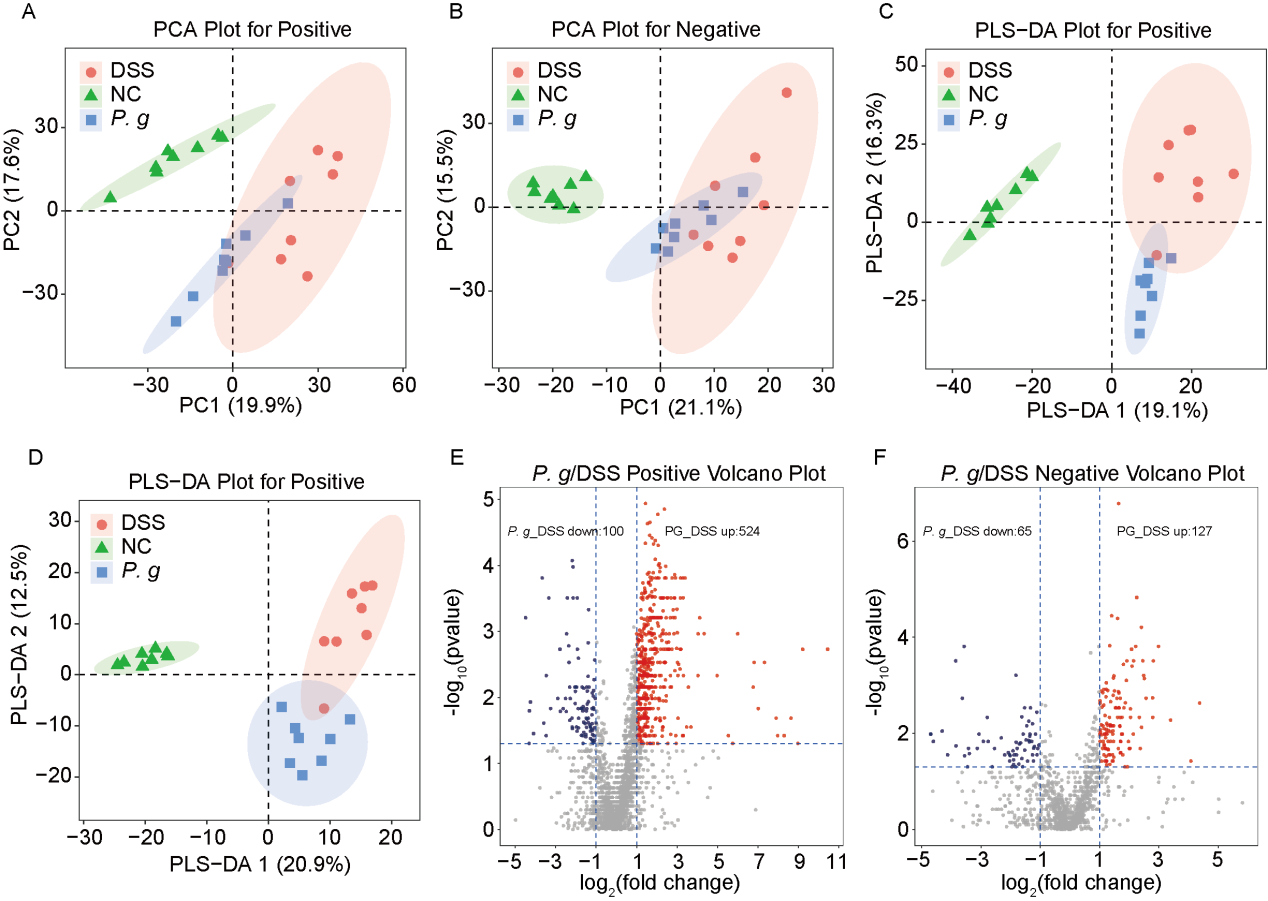


**Figure S8.** The influence of *P. goldsteinii* on the metabolites of gut microbiota. PCA analysis for metabolites of gut microbiota in positive (A) and negative (B) ion models. The PLS-DA analysis in positive (C) and negative (D) ion models. Volcanic map analysis of differential metabolites in positive (E) and negative (F) ion models. N=7.


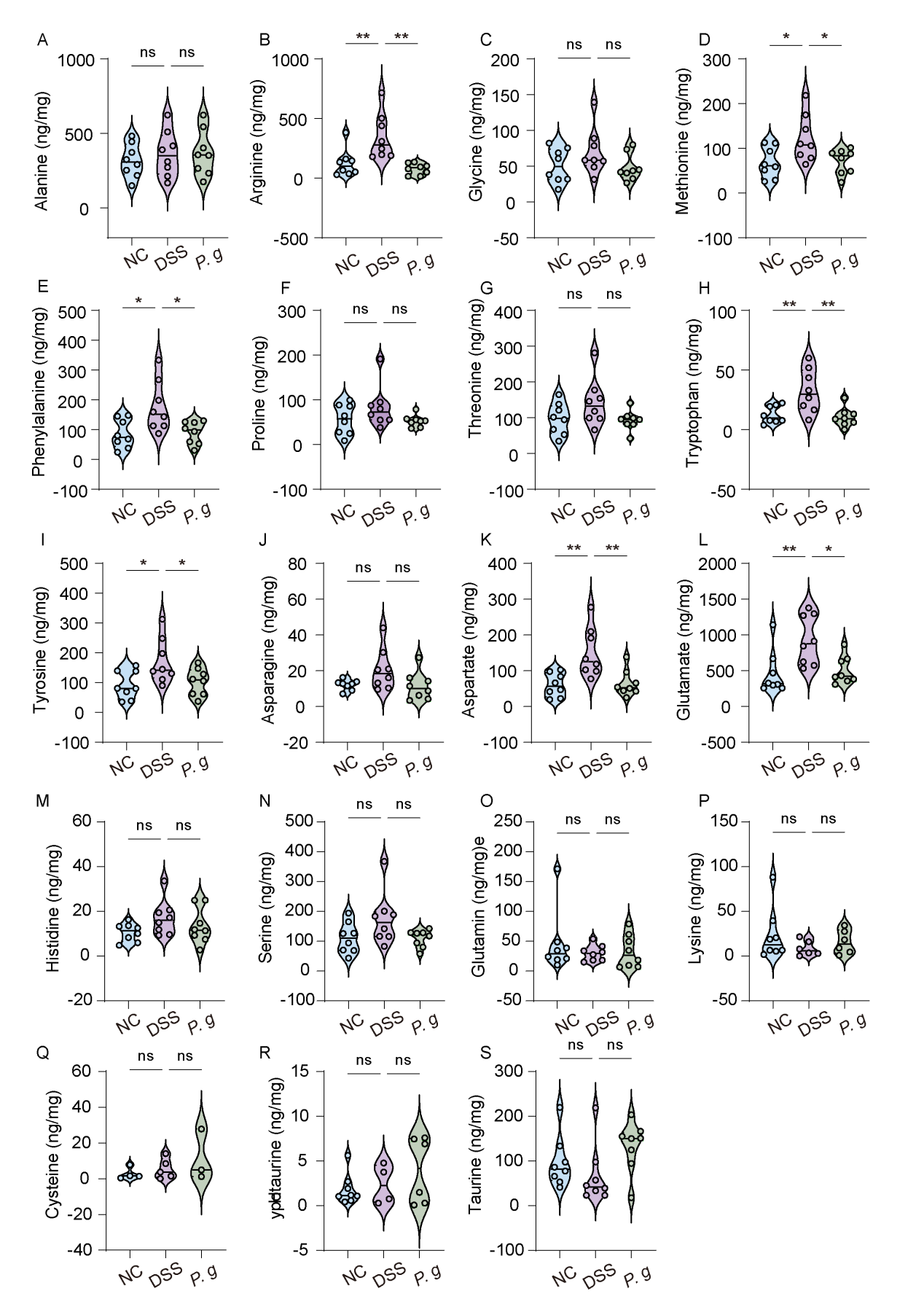


**Figure S9.** Effect of *P. goldsteinii* on amino acid metabolism. Data are presented as mean±SD. N=8. Compare with indicated group, *P<0.05, **P<0.01, ns: no significance.


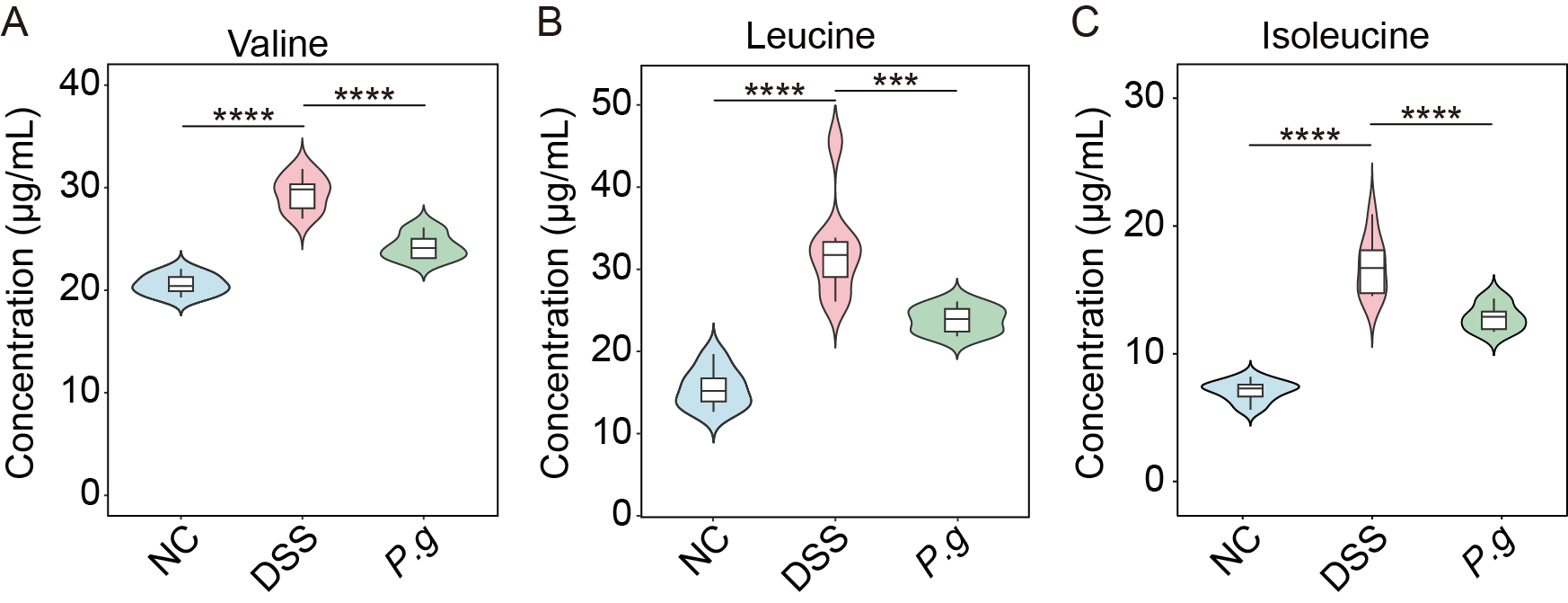


**Figure S10.** Effect of *P. goldsteinii* on amino acid metabolism in serum samples. Data are presented as mean±SD (n=8). Compare with indicated group, ***P<0.001, ****P<0.0001.


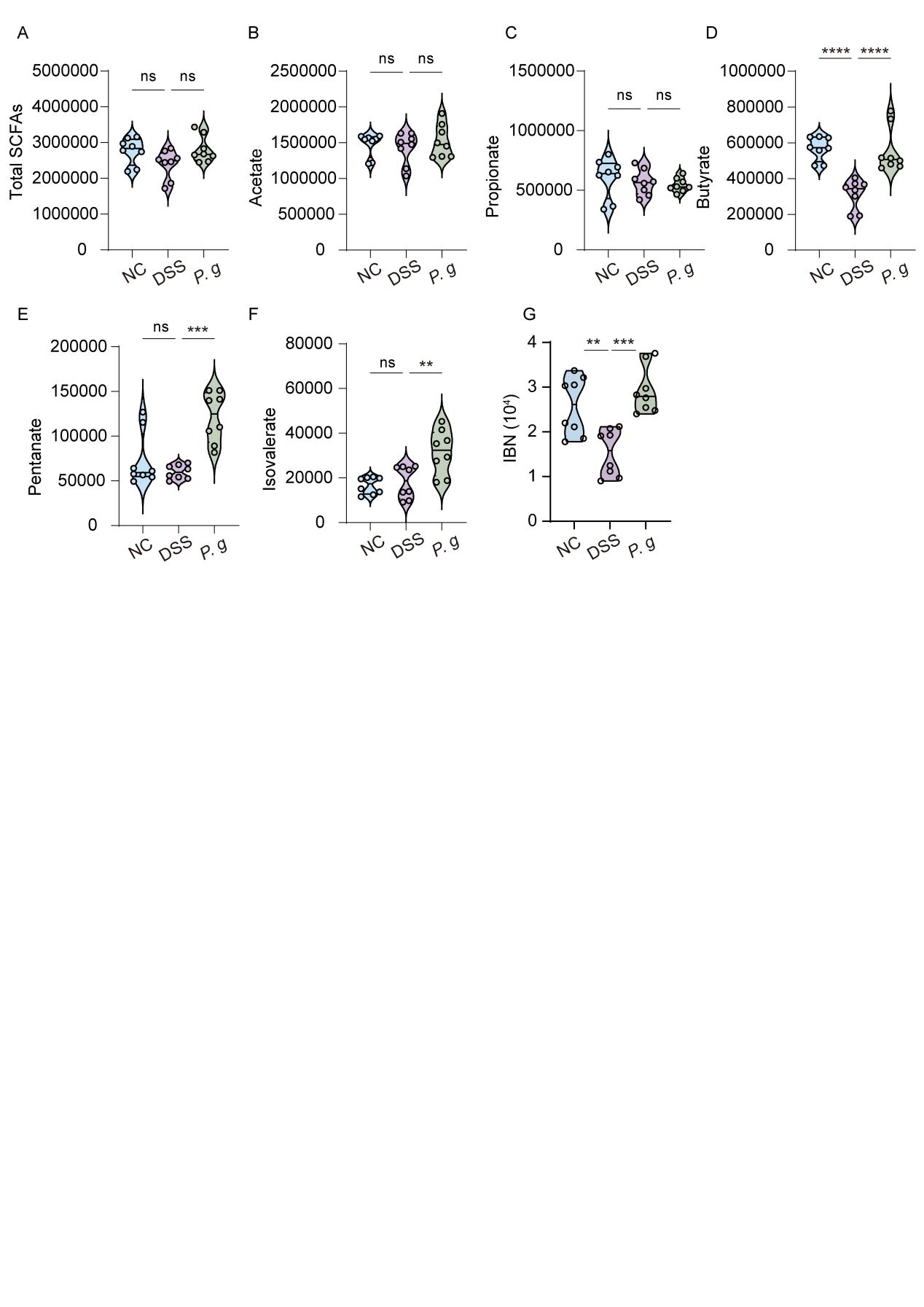


**Figure S11.** Effect of *P. goldsteinii* on SCFAs metabolism. The total SCFAs (A), Acetic acid (B), Propionic acid (C), Butyric acid (D), Pentanoic acid (E), Isovaleric acid (F) and IBN (G) in fecal samples were measured. N=8. Data are presented as mean±SD. Compare with indicated group, **P<0.01, ***P<0.001, **** P<0.0001, ns: no significance.


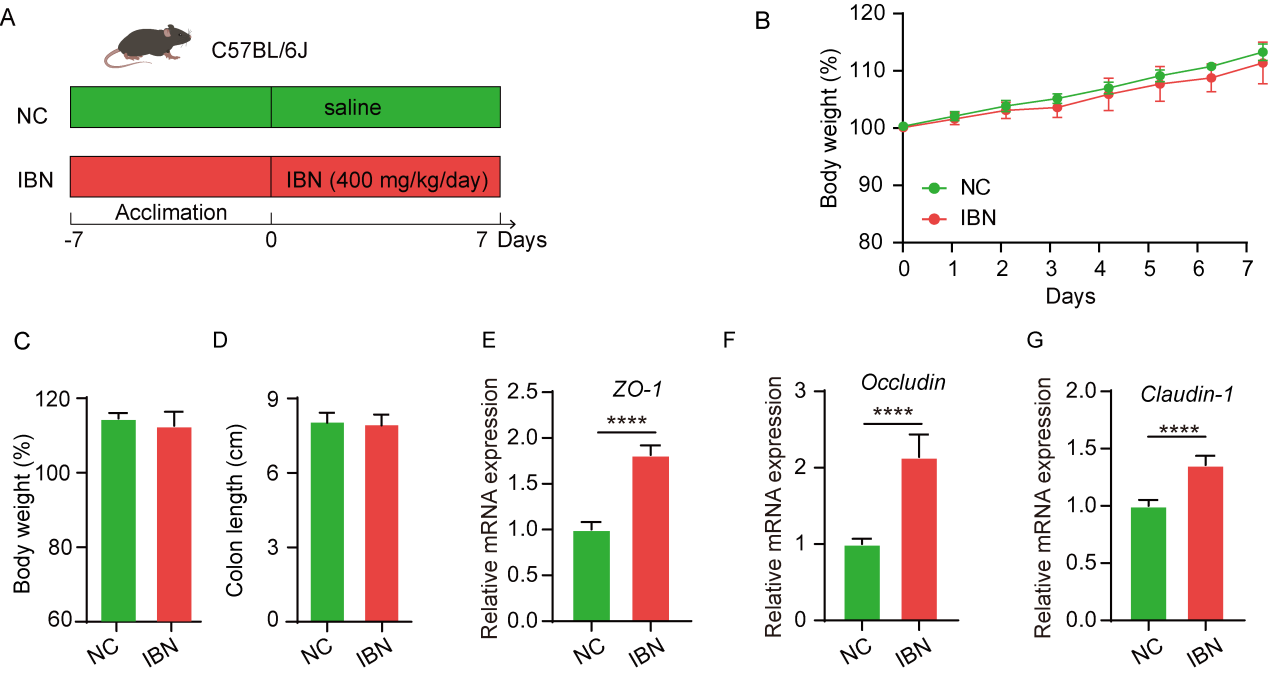


**Figure S12.** The effect of IBN for normal mice. (A) Animal Experiment Design. (B) The percentage of body weight change. (C) The final body weight change. (D) The colon length. N=8. The *ZO-1* (E), *Occludin* (F) and *Claudin-1* (G) in colonic tissues were measure using qRT-PCR (n=6). Data are presented as mean±SD. Compare with indicated group, ****P<0.0001.


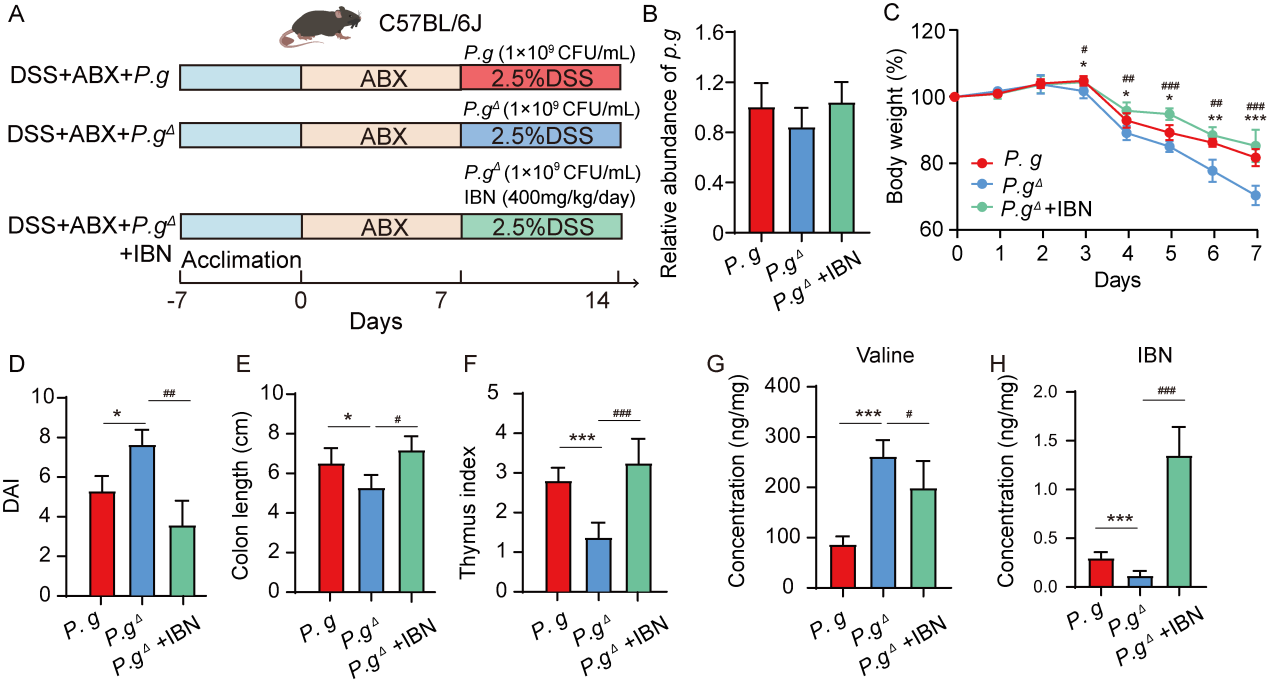


**Figure S13.** The anti-colitis effect of *P. goldsteinii^ΔiveE^* in supplementing IBN. (A) Animal Experiment Design. (B) The colonization analysis using qRT-PCR. (C) The percentage of body weight change. (D) DAI index. (E) The colon length. (F) The thymus index. The concentration of valine (G) and IBN (H) in fecal samples. N=8. Data are presented as mean±SD. Compare with *P. goldsteinii* and *P. g*^Δ^ group, *P<0.05, **P<0.01, ***P<0.001. Compare with *P. g*^Δ^ and *P. g*^Δ^ + IBN group, #P<0.05, ## P<0.01, ###P<0.001.

**
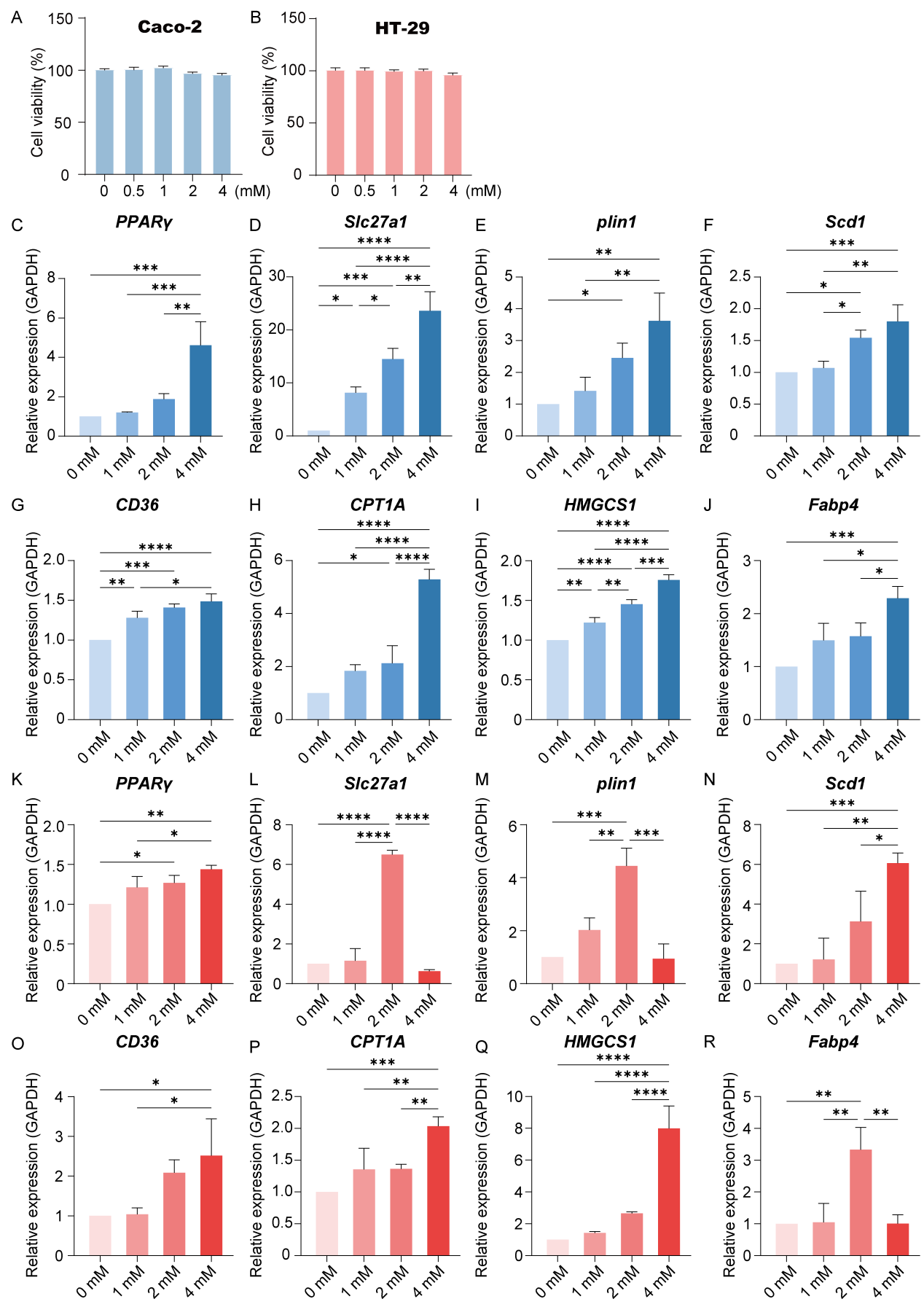
Figure S14.** The gene expression of PPARγ and its related genes for intestinal epithelial cell line. The viability of Caco-2 cell (A) and HT-29 cells (B) after IBN treatment. The gene expression of PPARγ and its related genes for Caco-2 cell (C-J) and HT-29 cells (K-R) using qRT-PCR. Cells with treatment of IBN (0, 1, 2, and 4 mM) for 24h. N=3. Data are presented as mean±SD. Compare with indicated group, *P<0.05, **P<0.01, ***P<0.001, ****P<0.0001.


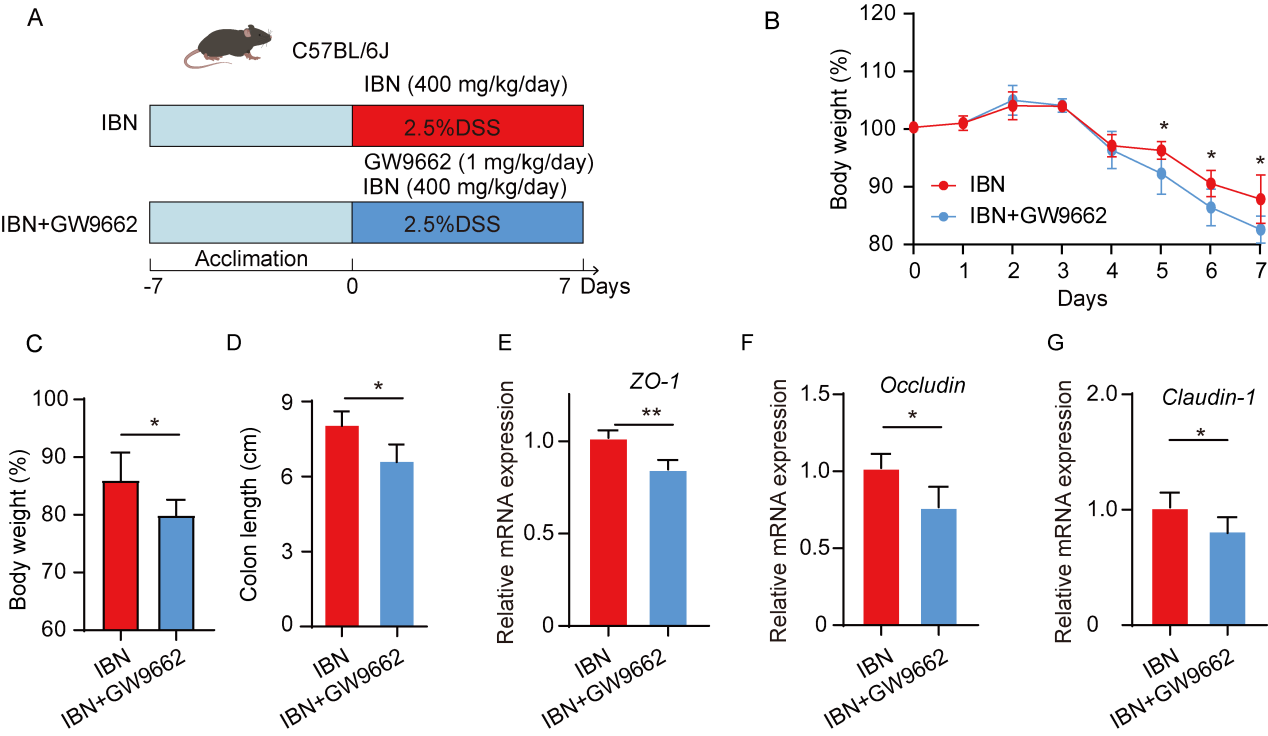


**Figure S15.** The effect of IBN and GW9662 for DSS induced colitis mice. (A) Animal Experiment Design. (B) The percentage of body weight change. (C) The final body weight change. (D) The colon length (n=8). The *ZO-1* (E), *Occludin* (F) and *Claudin-1* (G) in colonic tissues were measure using qRT-PCR (n=6). Data are presented as mean±SD. Compare with indicated group, *P<0.05, **P<001.
